# Commensal microbiota maintains alveolar macrophages with a low level of CCL24 production to generate anti-metastatic tumor activity

**DOI:** 10.1038/s41598-017-08264-8

**Published:** 2017-08-07

**Authors:** Min Cheng, Yongyan Chen, Liang Wang, Wen Chen, Ling Yang, Guodong Shen, Tingjuan Xu, Gan Shen, Zhigang Tian, Shilian Hu

**Affiliations:** 1 Gerontology Institute of Anhui Province, Anhui Province Hospital, Anhui Medical University, Hefei, 230001 China; 2 Anhui Provincial Key Laboratory of Tumor Immunotherapy and Nutrition Therapy, Hefei, 230001 China; 3 Institute of Immunology and The CAS Key Laboratory of Innate Immunity and Chronic Disease, School of Life Science and Medical Center, University of Science and Technology of China, Hefei, 230027 China; 4 School of Life Sciences, University of Science and Technology of China, Hefei, 230027 China

## Abstract

Microbiota maintains host tissue homeostasis and influences tissue-resident macrophages. However, the mechanisms by which commensal bacteria in regulating the alveolar macrophages remain unclear. Here, by using an antibiotic-treated (Abt) mouse model, we found commensal bacteria depletion induced lower frequencies and numbers of alveolar macrophages. This effect was accompanied by the altered levels of genes involved in several biological pathways, including M2 macrophage polarization, as determined by gene expression analysis. Alveolar macrophages from the Abt mice had higher protein and gene levels of Arg1, CCL24, IL-13, IL-10, IL-6 and IL-1β, which could be recovered to normal levels by reconstructing commensal bacteria in the upper respiratory of Abt mice. Moreover, alveolar macrophages performed significant enhancement of M2 functions, especially CCL24 secretion, in the Abt mice challenged with B16/F10 melanoma. Adoptive transfer of normal alveolar macrophages or antibody neutralization of CCL24 significantly recovered the decrease of γδT17 cells and rescued the defect anti-tumor response of Abt mice, indicating the elevated amount of alveolar macrophage-derived CCL24 inhibited γδT cell mediated anti-tumor response. In conclusion, we demonstrated the ability of commensal bacteria to maintain alveolar macrophages with a low level of CCL24 production, which was necessary for the normal anti-tumor response in the lung.

## Introduction

Enormous number of commensal microbiota reside on the surface of the body, including the skin, gastrointestinal, respiratory and urogenital tracts. The microbiota integrates into whole-organism physiology and maintains host tissue homeostasis. Disruption of this homeostasis would induce various disorders, such as infections, autoimmunity and autoinflammation, metabolic syndromes and cancer^1^. These inflammatory disorders are influenced by the interactions between the microbiota and the innate immune system including epithelial cells, myeloid cells, innate lymphoid cells (ILCs), dendritic cells, mast cells, etc.^1, 2^. In 2014, we reported that commensal microbiota can regulate the host immune surveillance of tumor cells in the lungs through γδT17 cell immunity^3^. However, the mechanisms underlying the interactions between microbiota and lung immune cells were not clearly described and deserved further investigation.

As reported, the biology of tissue-resident macrophages are strongly influenced by the microbiata such as in the central nervous system, skin, intestine and lungs^5–7^. Neonatal alveolar macrophages showed an early unresponsiveness to *Pneumocystis* infection, which is both intrinsic and related to the immunosuppressive environment in neonatal lungs^8^. When exposed to a mixture of microbial extracts early in life, the phagocytic and intracellular killing activities of alveolar macrophages against *S*. *pneumoniae* were enhanced in newborn mice^9^. As the mice grow after birth, commensal microbiota of the mucosal tissue is gradually established and improved, necessarily regulating the local immune microenvironment including the airway. The phenotypes and functions of alveolar macrophages are considerably influenced by the airway microenvironment^4^.

In response to environment-derived signals, macrophages polarize and acquire different functional properties. M1 macrophages (induced by exposure to interferon-γ (IFN-γ) and lipopolysaccharide (LPS)) exert potentiated cytotoxic and anti-tumor properties, whereas M2a macrophages (induced by interleukin (IL)-4 and IL-13) and M2b macrophages (induced by combined exposure to immune complexes and Toll-like receptor (TLR) or IL-1R agonists) exert immunoregulatory functions and drive Th2 activation, and M2c macrophages (induced by IL-10) are involved in suppression of immune responses and tissue remodeling^10^. Antibiotic treatment promoted fungal overgrowth in the gut, which increased plasma prostaglandin E2 (PGE2) levels. PGE2 contributed to the increased expression of arg1, chi3l3, and retnla (markers of alternatively activated M2 macrophages) in alveolar macrophages and promoted allergic airway inflammation^6^. Additionally, the gut microbiota enhanced the ability of primary alveolar macrophages to phagocytose *Streptococcus pneumoniae* and contributed to pulmonary bacterial clearance^11^. Evidence also indicated that commensal bacteria in the respiratory tracts may provide protection against viral infection through macrophages in the lungs. Bacterial colonization of *Staphylococcus aureus* recruited peripheral monocytes into the alveoli, which polarized to M2 alveolar macrophages in this environment, and dampened influenza-mediated acute lung injury^12^. Macrophages are originally thought to show anti-tumor activities, but clinical and experimental data suggest that macrophages promote tumor initiation and malignant progression in the large majority of cases^13, 14^. The role of commensal bacteria in regulating the mucosal macrophages, such as those in the lungs, during the immune surveillance of tumor cells remains unclear.

CCL24 (eotaxin-2), is selectively induced in M2a macrophages, which consequently recruit eosinophils, basophils and Th2 cells through the CCR3 receptor^10, 15^. CCL24 played significant roles in perpetuating allergic inflammation and the subsequent development of the late cutaneous reaction *via* eosinophil recruitment^16–19^. High levels of CCL24 were also observed and strongly associated with the primary colorectal and liver metastatic tumors, but with no recruitment of lymphocytes^20^. Thus, the role of macrophage-derived CCL24, particularly in tumor development, is worthy of attention. In this study, we used an antibiotic-treated (Abt) mouse model and firstly demonstrated that commensal microbiota maintained alveolar macrophages with a low level of CCL24 production, which was necessary for them to generate anti-tumor activity.

## Materials and Methods

### Mice and antibiotics treatment

Four- to five-week-old female C57BL/6 mice were obtained from the Shanghai Experimental Center of the Chinese Science Academy (Shanghai, China). The Abt experimental groups were assigned as previously described^3^. The mice were treated for 4 to 6 weeks with ampicillin (1 g/L), vancomycin (0.5 g/L), neomycin sulfate (1 g/L) and metronidazole (1 g/L) in their drinking water, which was changed twice per week. All mice were maintained under specific-pathogen-free (SPF) and controlled conditions (22 °C, 55% humidity, and a 12 h day/night rhythm). The animal experiments were approved by the Local Ethics Committee for Animal Care and Use at Anhui Medical University and were conducted in accordance with the Guide for the Care and Use of Laboratory Animals granted by Anhui Medical University.

### Assessment of B16/F10 lung melanoma

B16/F10 cells (a mouse melanoma cell line) were obtained from the American Type Culture Collection (ATCC, USA) and maintained in DMEM (Gibco BRL, USA) containing 10% heat-inactivated fetal bovine serum (FBS) (ExCell Biology, Shanghai, China). Mice were injected intravenously (i.v.) with 1 × 10^5^ B16/F10 cells. On day 17 after tumor introduction, the mice were euthanized, and the metastatic lung foci were counted. All lung lobes were evaluated.

### Isolation of lung mononuclear cells (MNCs)

As previously described^21^, MNCs were isolated from the lungs *via* density gradient centrifugation with 40% and 70% Percoll.

### Flow cytometry analysis

As previously described^3^, for the surface phenotype assay, 1 × 10^6^ cells were blocked with 10 μl rat serum for 30 min at 4 °C and then stained with the indicated antibody for 30 min at 4 °C in the dark. For the intracellular cytokine assay, the cells were stimulated with PMA (Sigma, St Louis, MO), monensin (Sigma, St Louis, MO) and ionomycin (Calbiochem, San Diego, CA, USA) for 4 h. The cells were labeled for surface markers, fixed, permeabilized, and then labeled with the indicated intracellular antibody for 30 min at 4 °C in the dark. All data were acquired using a FACS-Verse flow cytometer (Becton-Dickinson, Franklin Lakes, NJ, USA) and analyzed using FlowJo 7.6.1 software. For alveolar macrophages, all lung MNCs were gated by FSC and SSC. The monoclonal antibodies for FACS are shown in Supplemental Table 1.

### Purification of alveolar macrophages

Isolated lung MNCs were stained and then alveolar macrophages (F4/80^hi^ CD11c^hi^) were purified by using a FACS Aria II flow cytometer (Becton Dickinson, Franklin Lakes, NJ, USA). The separated cells were >95% pure.

### Immunofluorescent staining

For immunofluorescent staining of lung samples, 10 μm sections were submerged in acetone for 15 min, then tissues were blocked for 30 min and subsequently incubated with a 1:100 dilution of an anti-mouse F4/80 antibody (123101, clone BM8, Biolegend, San Diego, CA) and an anti-mouse CD11c antibody (117301, clone N418, Biolegend, San Diego, CA) overnight at 4 °C. After washing with PBS, the sections were incubated for 30 min with a goat anti-rat IgG (H + L) antibody (ZF-0318, ZSGB-BIOTECH CO., LTD, Beijing, China) and a goat anti-Armenian Hamster IgG (H&L) antibody (Alexa Fluor 488, ab173003, Abcam, UK). After washing again with PBS, the tissues were stained with DAPI (C1005, Beyotime, Beijing, China) for 5 min. Then, the sections were washed twice with PBS, and the stained tissues were imaged using a Leica SP5 laser-scanning confocal microscope (Leica Microsystems, Wetzlar, Germany).

### Microarray analysis

Total RNA was extracted and purified from sorted alveolar macrophages (F4/80^hi^ CD11c^hi^) using a miRNeasy Mini Kit (217004, QIAGEN, GmBH, Germany) and a RIN number (RIN cutoff value: 7.0) was determined to assess RNA integration using an Agilent Bioanalyzer 2100 (Agilent technologies, Santa Clara, CA, USA). Microarray experiments were performed by Shanghai Biotechnology Corporation (Shanghai, China). Total RNA was amplified and labeled using a Low Input Quick Amp WT Labeling Kit (5190–2943, Agilent technologies, Santa Clara, CA, USA). The labeled cRNA was purified using an RNeasy Mini Kit (74106, QIAGEN, GmBH, Germany). Each slide was hybridized with 1.65 μg Cy3-labeled cRNA using a Gene Expression Hybridization Kit (5188–5242, Agilent technologies, Santa Clara, CA, USA) in Hybridization Oven (G2545A, Agilent technologies, Santa Clara, CA, USA). After 17 h of hybridization, slides were washed in staining dishes (121, Thermo Shandon, Waltham, MA, USA) with a Gene Expression Wash Buffer Kit (5188–5327, Agilent Technologies, Santa Clara, CA, USA). Slides were scanned using an Agilent Microarray Scanner (G2565CA, Agilent Technologies, Santa Clara, CA, US) with default settings, Dye channel: Green, Scan resolution = 3 μm, PMT 100%, 20 bit. Data were extracted with Feature Extraction software 10.7 (Agilent Technologies, Santa Clara, CA, US). The raw data were normalized with a quantile algorithm using GeneSpring Software 11.0 (Agilent Technologies, Santa Clara, CA, USA). PCA analysis is performed by use of ClustVis^22^, Pareto scaling is applied to rows; SVD with imputation is used to calculate principal components. Each sample is the sorted alveolar macrophages from 15 mice. The microarray analysis was completely repeated for the reliability.

### Hierarchical clustering

Hierarchical clustering was performed by using freeware TM4-MEV software^23^. Data underwent Z-score normalization to facilitate the direct comparison of parameters with vastly different ranges. Briefly the average and standard deviation (SD) of the data for each parameter were calculated across groups. The Z-score was then calculated as the average for each data group subtracted from each data point and the difference divided by the SD for each data group (X- Avg)/SD. Hierarchal clustering (the average linkage clustering using the Euclidean distance as the distance metric) was performed within the parameters^24^.

### Gene Ontology (GO) and Kyoto Encyclopedia of Genes and Genome (KEGG) pathway analysis

To better understand the large number of DEGs in the microarray data and to identify specific signaling pathways, GO and KEGG pathway analysis were performed as previously described^25, 26^. DEGs (FC ≥ 2) were converted to Entrez-IDs for GO and KEGG analysis with R 3.2.3 using the library GO stats 2.34.0 and the R Bioconductor genomewide mouse annotations from package org.Mm.eg.db (version 3.3.0). The results were sorted by the p-value (p < 0.05).

### Quantitative real-time PCR

Total cellular RNA was extracted and purified from the sorted alveolar macrophages (F4/80^hi^ CD11c^hi^) using a miRNeasy Mini Kit (217004, QIAGEN, GmBH, Germany). The process was performed as described in the Supplemental Materials and Methods. Gene expression levels were quantified using the ΔΔCt method. Information on gene-specific primers is included in Supplemental Table 2.

### Blood agar plate (BAP) culture

100 mg fresh stool was dissolved in 1 ml PBS, and then serially diluted. Dilutions (10^−6^, 10^−7^, 10^−8^) of 100 μl volume were coated on the blood agar plates (9 cm, Hefei Tianda Diagnostic Reagent, Co., Ltd.). To quantify the commensal bacteria burden in the upper respiratory tract, nasal washes were collected by back-flushing 2 ml sterile saline from the posterior nasopharyngeal opening, and then 100 µl lavage fluid was dissolved in 1 ml PBS for serial dilutions^27^. Dilutions (10^−2^, 10^−3^, 10^−4^) of 100 μl volume were coated on the blood agar plates (9 cm, Hefei Tianda Diagnostic Reagent, Co., Ltd.). After 36 h culture in air incubator at 37 °C, CFU of bacteria were measured.

### Bacterial diversity analysis

Nasal wash and stool samples were collected and stored at −80 °C before use. DNA was extracted from nasal wash by using a DNeasy Blood & Tissue Kit (69504, QIAGEN, GmBH, Germany) and Lysozyme from chicken egg white (L6876, Sigma, St Louis, MO). DNA was extracted from 0.18–0.22 g stool using a QIA amp DNA Stool Mini Kit (51504, QIAGEN, GmBH, Germany). The DNA was recovered with 30 µL of AE buffer (Qiagen). The 16S ribosomal RNA (rRNA) gene was analyzed to evaluate the bacterial diversity by using Illumina Miseq (Novogene Bioinformatics Technology Co., Ltd, Beijing, China). 16S V3-4 area was selected for sequencing which is performed with Hiseq. 2500 PE250 (pair-end 250 bp). 16S genes were amplified by using the specific primers (341F: 5′-CCTAYGGGRBGCASCAG-3′; 806R:5′-GGACTACNNGGGTATCTAAT-3′) with the barcode placed at the 5′end. Paired-end reads were assigned to each sample according to the unique barcodes. Sequences were analyzed using QIIME software package. First, reads were filtered by QIIME quality filters; then in-house Python scripts were developed to pick operational taxonomic units (OTUs) by making OUT table. Sequences with ≥97% similarity were assigned to the same OTUs. Representative sequences were chosen for each OTU and use the RDP classifier to annotate taxonomic information for each representative sequence. In order to compute Alpha Diversity, the OTU table was rarified and three metrics were calculated: Chao1 estimates the species abundance; Observed Species estimates the amount of unique OTUs found in each sample, and Shannon index. Rarefaction curves were generated based on these three metrics. QIIME calculates both weighted and unweighted unifrac, which are phylogenetic measures of beta diversity.

### Cytokine detection

Purified alveolar macrophages (3 × 10^5^ cells/well) were stimulated with 1 µg/ml LPS (L2654, Sigma, St Louis, MO), 100 µg/ml PolyI:C (P1530, Sigma, St Louis, MO), 1 µg/ml flagellin (40067-V07E, Sino Biological Inc.) or 20 ng/ml PMA (P1585, Sigma, St Louis, MO) with a total volume of 600 µl DMEM supplemented with 10% fetal bovine serum for 48 h. Cytokines in the macrophage culture supernatants were detected. Eotaxin-2 (CCL24) (ab100681, Abcam, UK) and Arg I (KB13668, Shanghai Jiang Lai Biotechnology Co., China) were detected by ELISA. IL-6 (558301), IL-1β (560232), IL-10 (558300) and IL-13 (558349) were detected by Flow CBA Kit (BD Pharmingen, San Diego, CA, USA).

### Adoptive transfer of alveolar macrophages

As previously described^12, 28^, purified alveolar macrophages (2 × 10^5^ cells/mouse in 50 μl PBS) from the normal 9- to 10-week-old female C57BL/6 mice were adoptively transferred intranasally (i.n.) into the Abt mice one day before the B16/F10 challenge, and additional transfers were performed every week. The same volume of PBS alone was used as the control. The experimental mice were anesthetized intraperitoneally (i.p.) with sodium pentobarbital (50 μg/g of body weight) before nasal dripping.

### Antibody neutralization

A recombinant anti-mCCL24 antibody (MAB528, R&D, Abingdon, UK) was injected i.p. into the Abt mice (100 μg/mouse in 250 μl of PBS) one day before B16/F10 challenge, and additional injections were performed every week. The same volume of PBS alone was used as the control.

### Statistical analysis

All data are shown as the mean ± SEM. Differences between the individual data were analyzed using Student’s *t*-test, analysis of variance (one-way ANOVA) or Wilcox test when appropriate. Least significant difference tests (LSD, 0 < α < 1) were used for the post hoc tests. A p-value < 0.05 was considered statistically significant.

## Results

### Alveolar macrophage number and gene expression are altered in Abt mice

**Figure 1:**
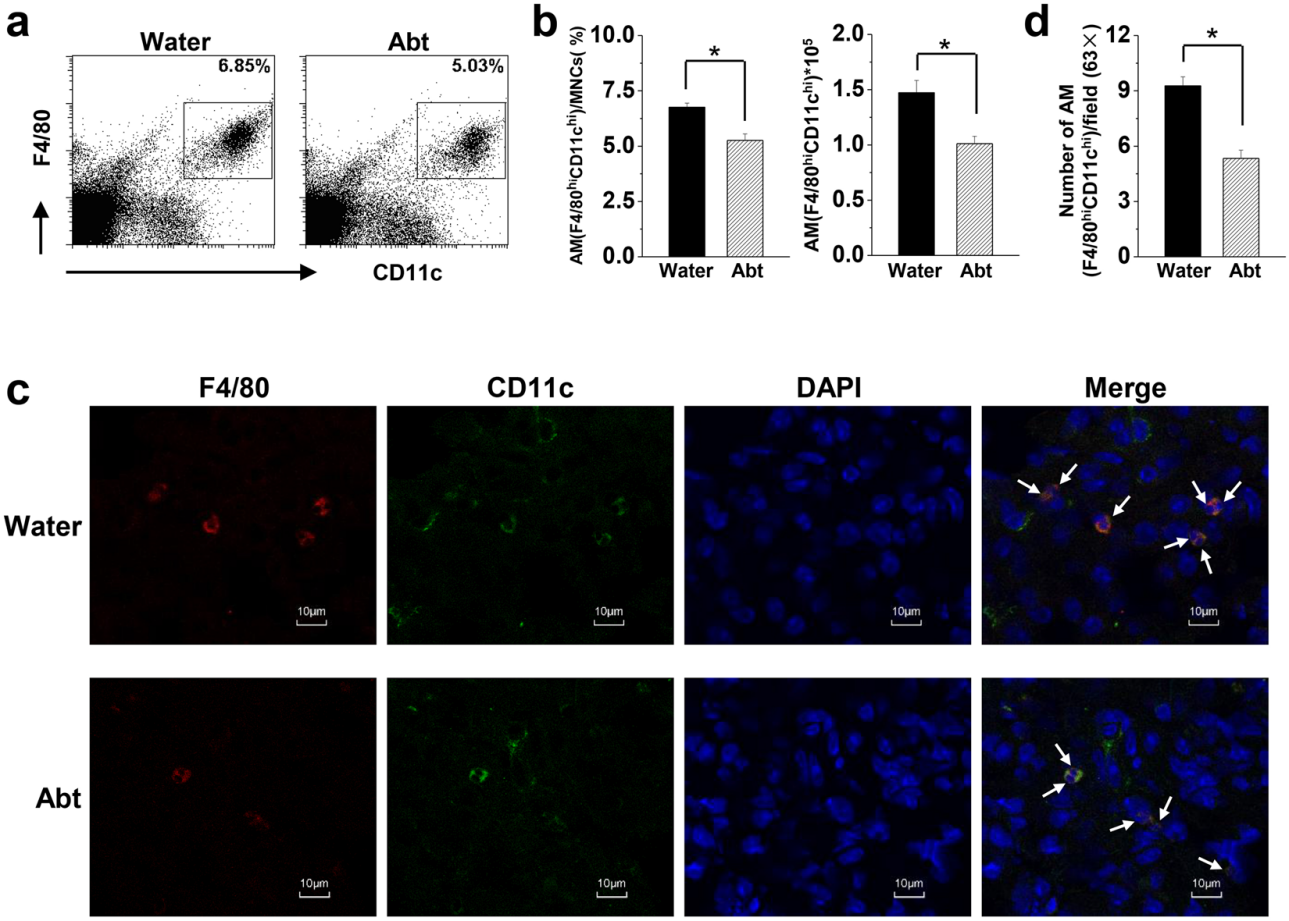
Decreased numbers of alveolar macrophages in Abt mice. C57BL/6 mice in the Abt group were given 1 g/L ampicillin, 0.5 g/L vancomycin, 1 g/L neomycin sulfate and 1 g/L metronidazole in their drinking water for five weeks. (**a**) Detection of alveolar macrophages (F4/80^hi^ CD11c^hi^) in mouse lung MNCs by FACS. (**b**) Frequency (alveolar macrophages/lung mononuclear cells) and number of alveolar macrophages (F4/80^hi^ CD11c^hi^) were analyzed. The MNCs were gated by FSC and SSC. (**c**) Immunofluorescence analysis of alveolar macrophages (F4/80^hi^ CD11c^hi^), indicated by arrows (magnification × 63). (**d**) F4/80^hi^ CD11c^hi^ cell numbers were counted and analyzed in 50 fields (63×) per tissue slide. There were five mice in each group. Data are shown as mean ± SEM. *p < 0.05 compared to the control group.

Commensal bacteria shape host immunity by interacting with epithelial barriers and stimulating a series of immune cells^29^. After five weeks of antibiotics treatment, the frequency and number of macrophages were significantly lower in the lungs of the Abt mice than in the lungs of the controls (Fig. 1a and b), and these were confirmed by immunofluorescence analysis (Fig. 1c and d).

**Figure 2:**
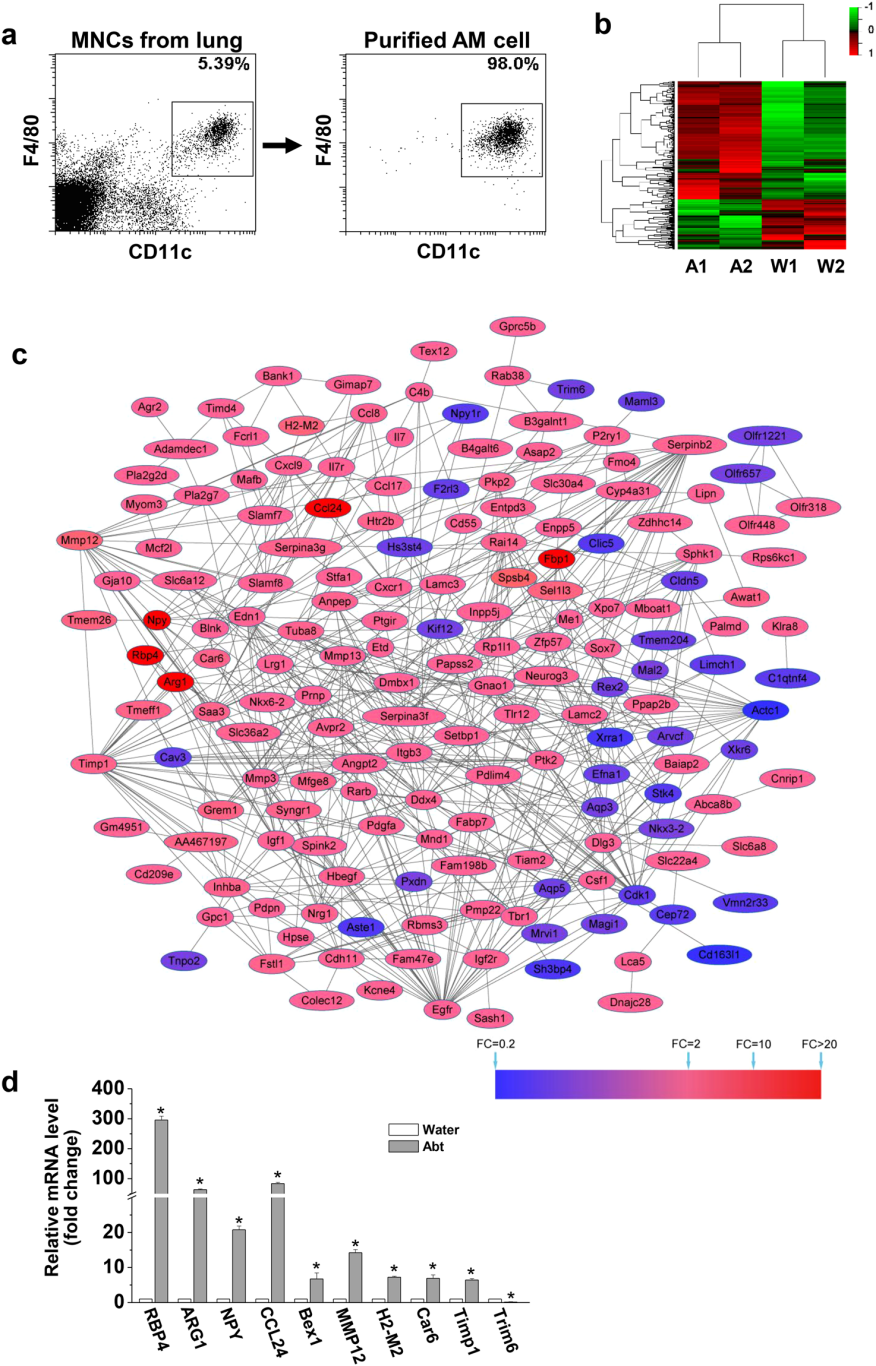
Altered gene expression in alveolar macrophages isolated from Abt mice. The mice in Abt group were given antibiotics for five weeks. Purified alveolar macrophages (F4/80^hi^ CD11c^hi^) were analyzed by GeneChip. (**a**) Alveolar macrophages (F4/80^hi^ CD11c^hi^) were sorted from lung MNCs. (**b**) The heatmap showed the differential expression of the indicated genes between the Abt group and the control group. DEGs were determined by Limma, and were row-based median normalized (2 samples/group, 15 mice/sample). (**c**) Protein-protein interaction (PPI) networks of the DEGs identified in the Abt group compared with the control. The red nodes indicate the up-regulated DEGs and the blue nodes indicate the down-regulated DEGs. Proteins associated with each other are linked by an edge. The degree of color represents the DEG fold change in reference to the scaleplate. (**d**) The mRNA expression levels of selected DEGs in the sorted macrophage cells (F4/80^hi^ CD11c^hi^) were measured by real-time PCR (n = 3). *p < 0.05 compared with the control group.

Then, purified alveolar macrophages (98% purity) were acquired for gene analysis (Fig. 2a). Principal component analysis (PCA) revealed good homogeneity of the two repeated samples in each group for a transcriptome study, with PC1 and PC2 accounting for 39.7% and 31.1% of the variance respectively (Supplemental Fig. 1a). Hierarchical clustering indicated favorable repeatability of the two samples in each group (Supplemental Fig. 1b). A total of 39430 genes were analyzed in alveolar macrophages to compare the Abt mice and the controls. Among 37401 positive signal values, 37091 were not altered in the Abt mice compared with the controls, whereas a greater than 2-fold change was detected for 310 expressed genes. Of these 310 DEGs, 249 genes in both Abt mice and the controls gave signal values that were distinguished from the background. Additionally, 246 genes had a known gene symbol and title, and 246 were annotated (Fig. 2b and Supplemental Fig. 1c). Based on these results, antibiotics treatment resulted in the differential expression of a large number of genes in alveolar macrophages.

### Classification of differentially modulated genes in alveolar macrophages according to their biological functions in Abt mice

There were 192 upregulated genes (78.05%) and 54 downregulated genes (21.95%) in the Abt group compared with the controls with a total of these 246 DEGs (≥2 fold change) (Supplemental Fig. 1d). Among these, 174 genes (139 upregulated genes and 35 downregulated genes) were involved in protein-protein interactions (Fig. 2c). The most important proteins (hub proteins) included Egfr, Timp1, Cdk1, Serpinb2, Mmp12, Mmp3, Ddx4, Mmp13, Itgb3, Ptk2, Edn1, Actc1, Dlg3, Inhba, Igf1, Anpep, Tuba8, Gnao1, Cxcl9, and Npy. All 154 DEGs with assigned biological functions were shown in Supplemental Table 3. Additionally, the top 10 altered genes were selected and detected by real-time PCR, and the results were consistent with the gene microarray data (Fig. 2d), indicating the significant difference in these gene expressions between the Abt mice and the controls.

**Figure 3:**
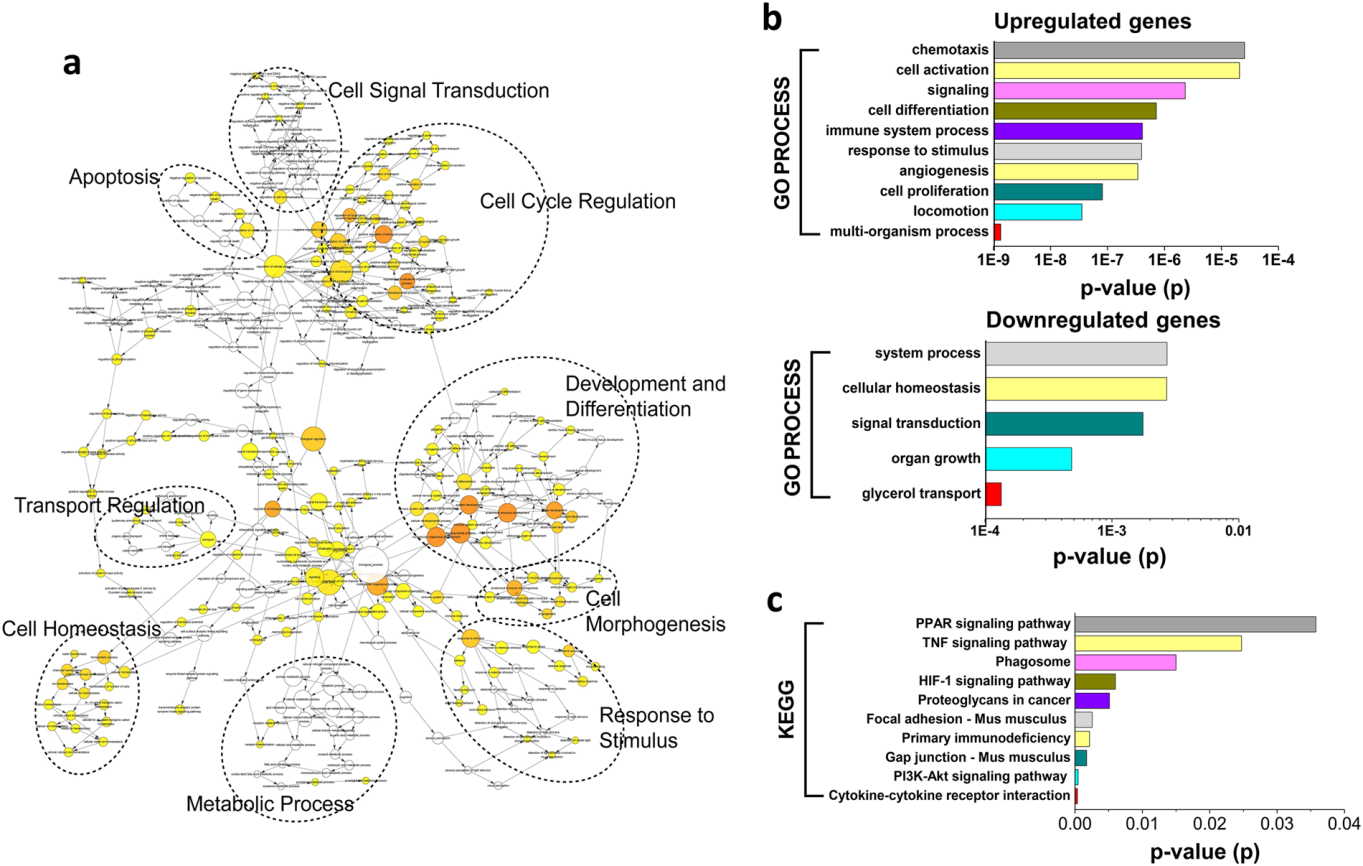
GO and KEGG analysis of differentially modulated genes in alveolar macrophages in Abt mice. The mice in Abt group were given antibiotics for five weeks. Purified alveolar macrophages (F4/80^hi^ CD11c^hi^) were analyzed by GeneChip. (**a**) An overview of the biological pathway analysis. BiNGO was used to categorize GO biological processes and generate a pathway enrichment network. (**b**) The list of DEGs was converted into Entrez-IDs for GO analysis with R 3.2.3 using the library GOstats 2.34.0 and the R Bioconductor genomewide mouse annotations from the package org.Mm.eg.db (version 3.3.0). (**c**) The list of DEGs was converted into Entrez-IDs for KEGG analysis with R 3.2.3 using the library GOstats 2.34.0 and the R Bioconductor genomewide mouse annotations from the package org.Mm.eg.db (version 3.3.0).

Further, BiNGO was applied to categorize the GO biological processes to assess the possible biological pathways in which the DEGs were involved. The pathway enrichment network showed the biological pathways including (1) cell signal transduction, (2) apoptosis, (3) cell cycle regulation, (4) development and differentiation, (5) transport regulation, (6) cell homeostasis, (7) cell morphogenesis, and (8) response to stimulus and metabolic process (Fig. 3a). Some GO terms were related to the immune processes, cell activation and chemotaxis, and other GO terms were related to cell proliferation and differentiation (Fig. 3b). According to the KEGG enrichment analysis, the DEGs were significantly enriched in 10 pathways, including cytokine-cytokine receptor interactions, the PI3K-Akt signaling pathway, Gap junctions and primary immunodeficiency (Fig. 3c).

### Alveolar macrophages prone to M2 are closely related to bacterial load and composition in the upper respiratory tract in Abt mice

**Figure 4:**
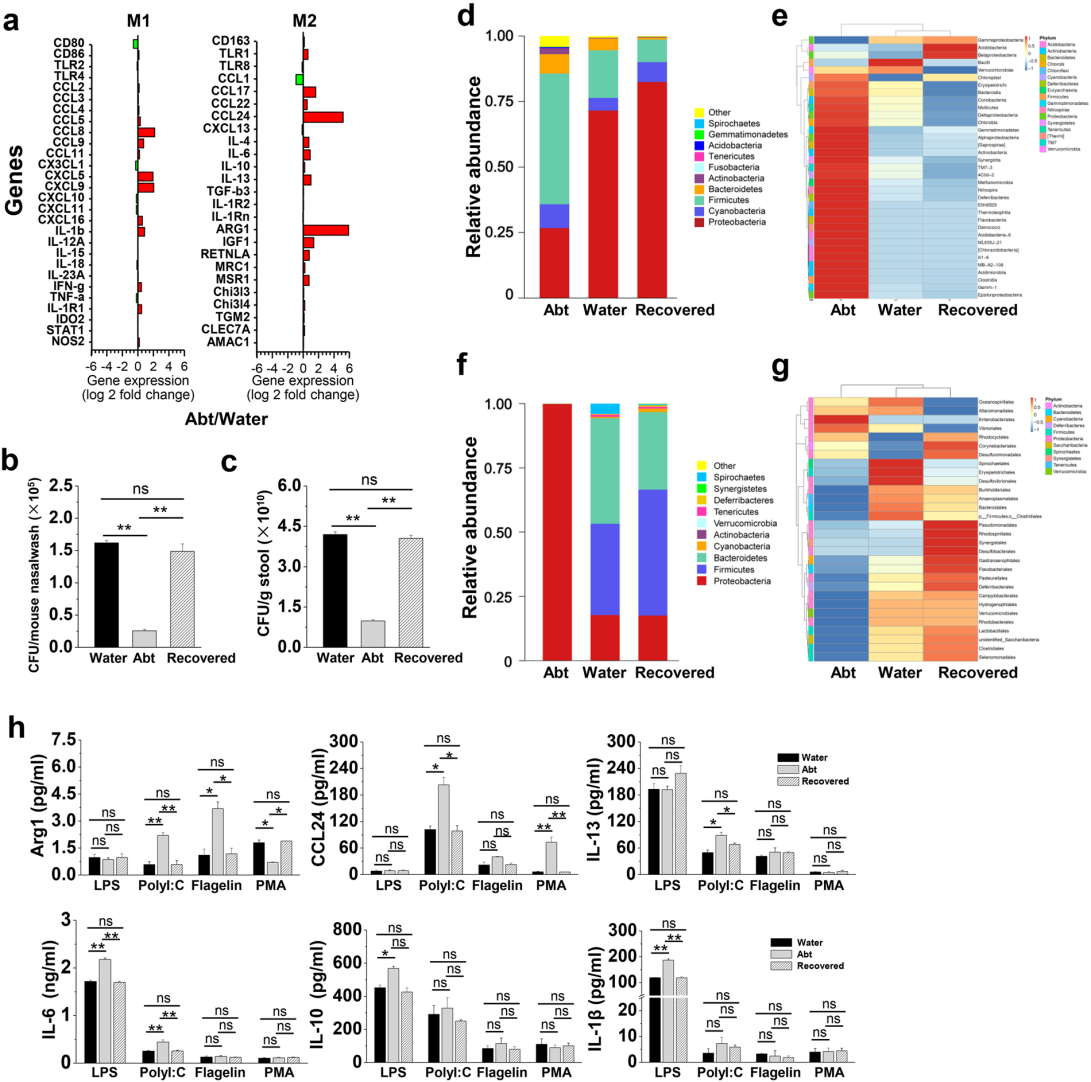
Recovered commensal bacteria restore the functions of alveolar macrophages in Abt mice. The mice in Abt group were given antibiotics for five weeks. In the recovered group, the mice were given antibiotics for five weeks and then co-housed with the wild type mice for four weeks. Purified alveolar macrophages (F4/80^hi^ CD11c^hi^) were analyzed. (**a**) M1 and M2 specific gene expression in alveolar macrophages from Abt mice was compared with that in control mice by GeneChip analysis. (**b** and **c**) Bacterial loads in the upper respiratory tract and stool from the recovered mice compared with those in the control mice (n = 3/group) were measured by BAP culture. (**d** and **e**) Relative abundances (**d**) and a clustering map (**e**) for the bacteria in the upper respiratory tract were determined by 16S rRNA analysis (3 samples/group, 10 mice/sample). (**f** and **g**) Relative abundances (**f**) and a clustering map (**g**) for the bacteria in the stool were determined by 16S rRNA analysis (3 mice/group). (**h**) Purified alveolar macrophages (F4/80^hi^ CD11c^hi^) were stimulated for 48 h in the indicated culture medium (1 µg/ml LPS, 100 µg/ml PolyI:C, 1 µg/ml flagellinor 20 ng/ml PMA) and then analyzed for the protein expression by ELISA (Arg1 and CCL24) and CBA (IL-13, IL-6, IL-10 and IL-1β) (n = 3). The data are shown as the mean ± SEM. *p < 0.05, **p < 0.01 compared with the control group.

The immune characteristics of alveolar macrophages isolated from the Abt mice and the controls were compared, and there were no significant differences in the expression of functional molecules MHC-II, CD86, CD127, CD69, CD16/32 and CCR7 (Supplemental Fig. 2). Genes specific to M1 and M2 macrophages were identified in alveolar macrophages based on the microarray data (Fig. 4a) and confirmed by PCR (Supplemental Fig. 3a and b), indicating that alveolar macrophages from the Abt mice exhibited both M1 and M2 phenotypic and functional molecules, but the M2 characteristics predominated, such as much higher expression levels of CCL24 and Arg1.

In the normal, the bacterial load in the upper respiratory tract was (1.62 ± 0.038) × 10^5^ CFU/mouse (Fig. 4b), which was much lower than that of (4.19 ± 0.113) × 10^10^ CFU/g in the stool (Fig. 4c). Antibiotics treatment resulted in a significantly decreased aerobic bacterial load (greater than 75%) in both the upper respiratory tract (Fig. 4b) and stool (Fig. 4c) in the Abt mice. We then performed a 16S rRNA assay to analyze the bacteria composition. In the upper respiratory tract, antibiotics treatment caused a change in the overall composition, and resulted in a low frequency of Proteobacteria and a high frequency of Firmicutes and Actinobacteria in the Abt mice (Fig. 4d and e). In the stool, antibiotics treatment also caused a change in the overall composition, with a high frequency of Proteobacteria and a low frequency of Firmicutes and Bacteroidetes in the Abt mice (Fig. 4f and g). When the Abt mice were co-housed with the water control mice for four weeks, the bacteria loads in the upper respiratory tract and stool recovered to the extent similar to the water control mice (Fig. 4b and c), and the bacterial composition in the upper respiratory tract and stool also recovered (Fig. 4d,e,f and g). In these recovered mice, alveolar macrophage functions were restored, as demonstrated by the reversed expression of Arg1, CCL24, IL-13, IL-10, IL-6 and IL-1β (Fig. 4h). These results indicated that alveolar macrophage functions were closely related to the bacterial load and composition in the upper respiratory tract.

### Alveolar macrophages inhibit anti-tumor immune responses through CCL24 in Abt mice

As we previously reported, commensal bacteria are essential for supporting the host immune response to cancer in the lungs^3^. Whether the altered alveolar macrophages involved in the cancer progression was further investigated.

**Figure 5:**
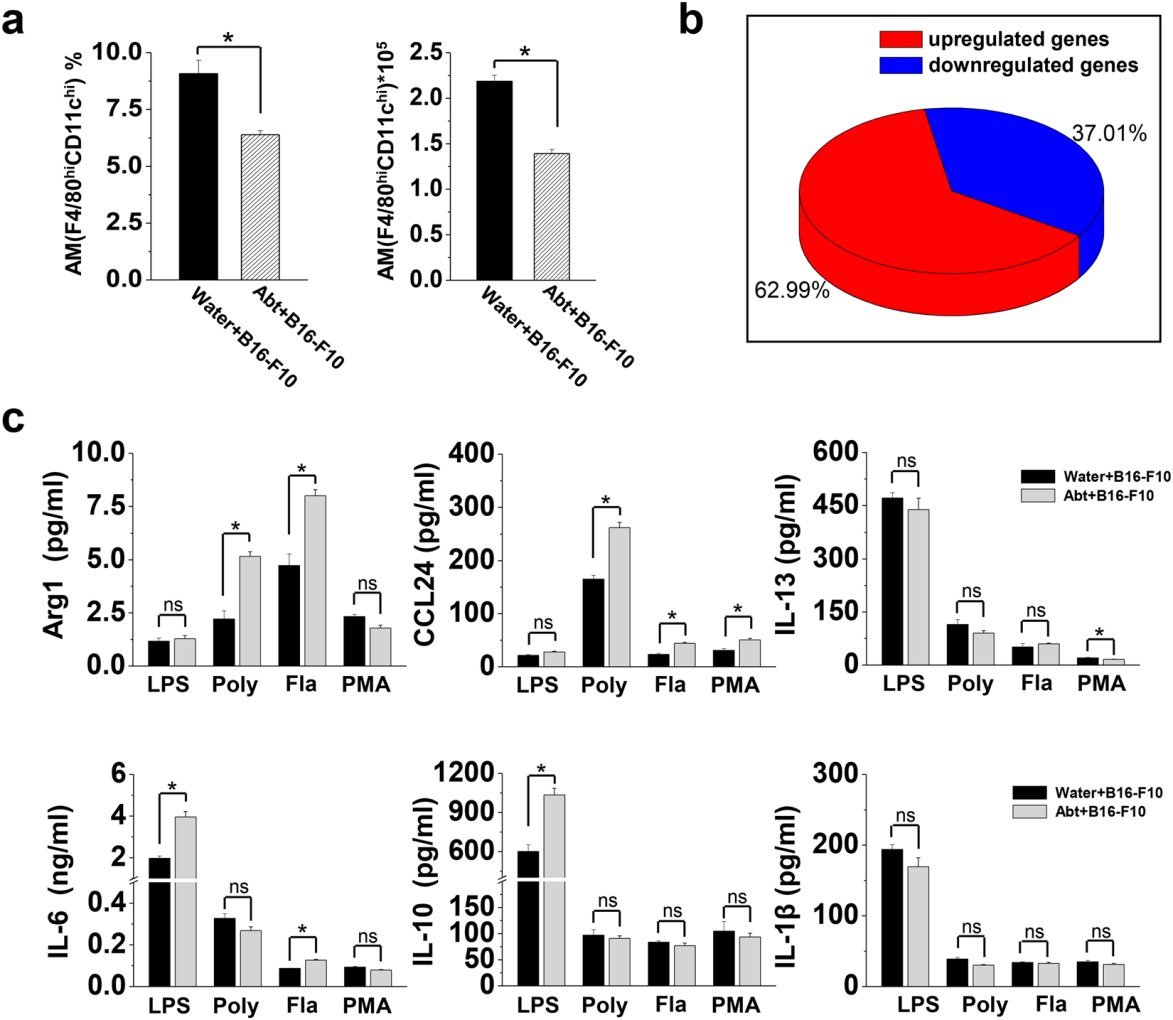
Decreased numbers but M2 polarization of alveolar macrophages in Abt mice challenged with B16/F10 melanoma. The mice were given antibiotics for five weeks and then challenged with B16/F10 cells (1 × 10^5^ cells/mouse, i.v.). On day 17 after the B16/F10 challenge, the lung MNCs were isolated. (**a**) Frequency and number of alveolar macrophages (F4/80^hi^ CD11c^hi^) in the lung MNCs from mice challenged with B16/F10 cells (n = 5). (**b**) Purified alveolar macrophages (F4/80^hi^ CD11c^hi^) were analyzed by GeneChip. The pie chart showed the distribution of DEGs in Abt group compared with the control. Unknown and duplicated genes were filtered for the analysis (2 samples/group, 15 mice/sample). (**c**) Purified alveolar macrophages (F4/80^hi^ CD11c^hi^) (3 × 10^5^ cells/well) were stimulated for 48 h in the DMEM containing 10% FBS with the stimulation of 1 µg/ml LPS, 100 µg/ml PolyI:C, 1 µg/ml flagellin or 20 ng/ml PMA, and then culture supernatants were analyzed for the cytokine expression by ELISA (Arg1 and CCL24) and CBA (IL-13, IL-6, IL-10 and IL-1β) (n = 3). The data are shown as the mean ± SEM. *p < 0.05 compared with the control group.

The frequencies and numbers of macrophages remained much lower in the lungs of the Abt mice 17 days after challenged with B16/F10 melanoma (Fig. 5a). Gene analysis revealed 80 upregulated genes (62.99%) and 47 downregulated genes (37.01%) (≥2 fold change) in the alveolar macrophages of the Abt mice after challenged with B16/F10 melanoma compared with the water mice challenged with B16/F10 melanoma (Fig. 5b). Among these 127 DEGs, 81 genes (54 upregulated genes and 27 downregulated genes) were found to undergo protein-protein interactions, particularly those genes related to the immune system processes and inflammatory responses (Supplemental Fig. 4a and b). These changes in gene expression resulted in a significant predominance of M2 cellular functions for the alveolar macrophages of the Abt mice, as demonstrated by the elevated expression levels of CCL24, IL-10, IL-6, Arg1 and IL-13 (Fig. 5c).

**Figure 6:**
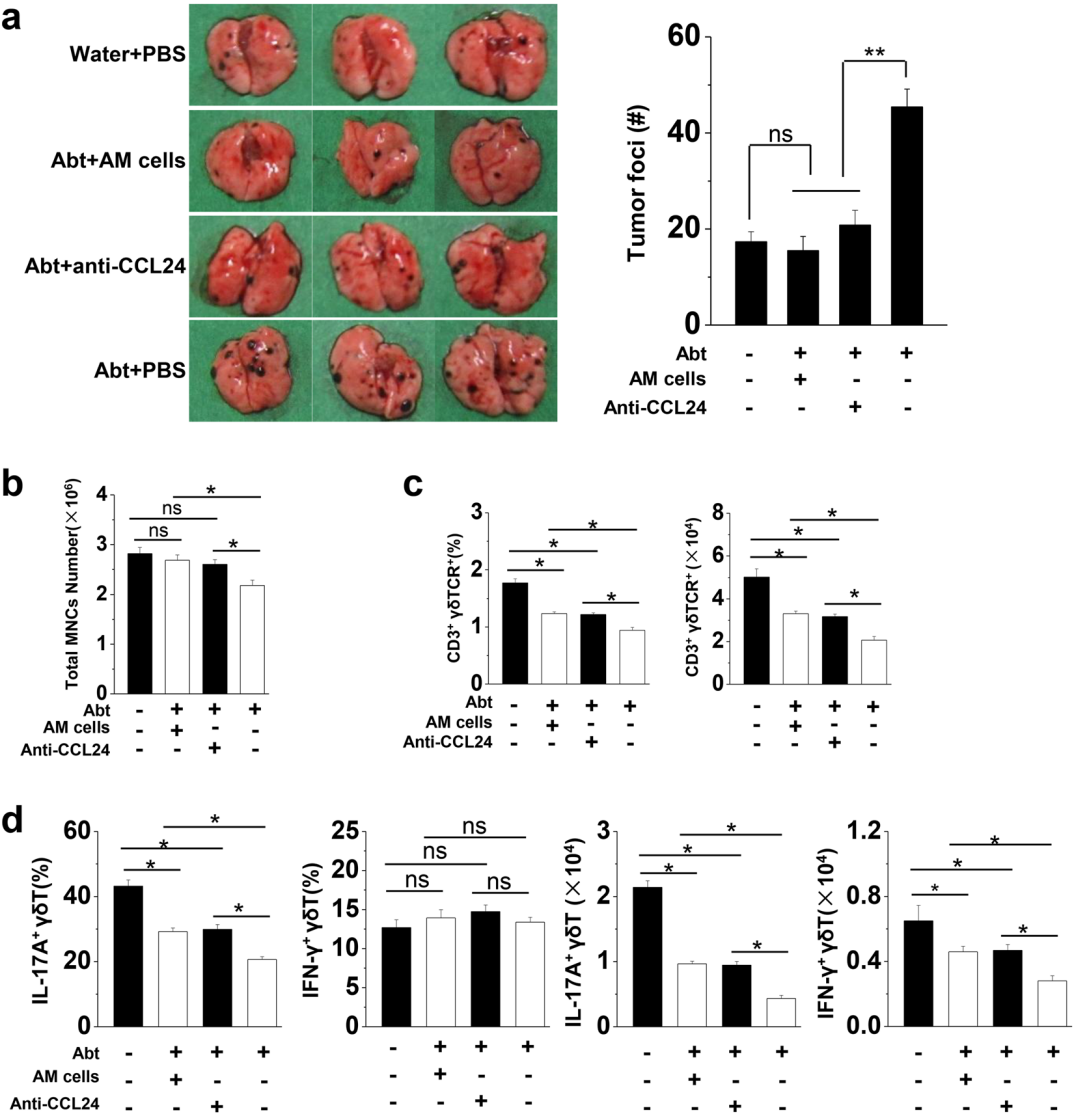
The adoptive transfer of normal alveolar macrophages or CCL24 neutralization rescues anti-tumor immune impairment in Abt mice. The mice were given antibiotics for five weeks and then challenged with B16/F10 cells (1 × 10^5^ cells/mouse, i.v.). Alveolar macrophages transfer and CCL24 neutralization were performed in the Abt mice. The mice were treated with antibiotics for the entire experimental period. (**a**) The lungs were analyzed on day 17 after the B16/F10 melanoma challenge. The graphs showed the total number of tumor colonies present in the lung lobes, and the numbers of tumor foci in the lungs were calculated. (**b**) The total number of MNCs in the lungs on day 17 after the B16/F10 challenge. (**c**) The frequency of γδT cells among lymphocytes in the lungs, and the absolute number were shown. The isolated MNCs were analyzed by FACS and the lymphocytes were gated by FSC and SSC. (**d**) The frequency of IL-17A^+^ γδT cells and IFN-γ^+^ γδT cells among total γδT cells in the lungs, and their absolute numbers were shown. There were six mice in each group. The data are shown as the mean ± SEM. *p < 0.05 compared with the control group.

Further, we purified alveolar macrophages from normal mice and adoptively transferred them (2 × 10^5^cells/mouse) into the Abt mice challenged with B16/F10 as described in Supplemental Fig. 5. As confirmed, (1.46 ± 0.074) × 10^5^ transferred macrophages (F4/80^hi^ CD11c^hi^ cells) could reach the lungs of the recipient mice (Supplemental Fig. 6). Remarkably, transferred alveolar macrophages resulted in smaller tumor size and less number of tumor foci in the lungs of the Abt mice on day 17 after the challenge with B16/F10 melanoma cells (Fig. 6a). These results were similar to those observed in PBS-treated water control mice, indicating normal alveolar macrophages were sufficient to restore the anti-tumor immune responses in the Abt mice. Furthermore, CCL24 neutralization also resulted in smaller tumor size and less number of tumor foci in the lungs of the Abt mice on day 17 after the challenge (Fig. 6a). These results indicated that the defective anti-tumor responses of the alveolar macrophages were dependent on their higher levels of CCL24 production in Abt mice.

Antibiotics treatment resulted in a decreased frequency of γδT cells and impaired IL-17A^ +^ γδT cells in the lungs after challenged with B16/F10 melanoma cells^3^. When the normal alveolar macrophages were transferred or the higher levels of CCL24 were neutralized, the frequency and number of γδT cells (Fig. 6b,c and Supplemental Fig. 7), particularly IL-17A^ +^ γδT cells (Fig. 6d), significantly increased in the lungs of the Abt mice after challenged with B16/F10 melanoma cells. This rescued phenomenon was observed in the lungs but not in the spleen (Supplemental Fig. 8).

## Discussion

In this study, we firstly demonstrated that commensal microbiota maintained alveolar macrophages with a low level of CCL24 production to generate anti-metastatic tumor activity. It has been reported that in the lung, the commensal microbiota enhanced early innate defenses against bacterial infection *via* Nod-like receptor ligands by inducing reactive oxygen species-mediated bacterial killing through alveolar macrophages^30^. Also, commensal microbiota primed the inflammatory response induced during lung ischemia reperfusion by inducing cytokine production of alveolar macrophages^31^. However, the mechanism by which the microbiota exerts its immune-modulating effects on alveolar macrophages is poorly understood.

Lots of evidence showed no existence of microbiota in the lower respiratory tract^32–36^. We also confirmed there is nonexistence of microbiota in the lungs of SPF wild type mice (data not shown). Thus, the nasal sample of upper respiratory tract was studied to explore the role of microbiota in lung immunity, as reported in the published^12^. It was found that the bacterial load in the upper respiratory tract was much lower than that in the stool (Fig. 4b and c), and the composition was different between the upper respiratory tract and stool (Fig. 4d and f). Oral antibiotics treatment is conventional to establish the commensal-depleted mice model to study the role of intestinal commensal microbiota^27, 37^. Our study revealed that after 5 weeks treatment of Abt (ampicillin, vancomycin, neomycin sulfate and metronidazole), the load of microbiota in mouse upper respiratory tract also remarkably decreased and the composition also altered significantly (Fig. 4b,d and e). And the alteration in the bacterial species was distinguished between the intestinal and the upper respiratory tract. The intact commensal microbiota in the upper respiratory tract was necessary to maintain the homeostasis of alveolar macrophages, including their frequency, number and functions (Fig. 1 and Supplemental Fig. 3). As reported in our previous study, the individual treatments with ampicillin, vancomycin, neomycin sulfate or metronidazole recapitulated the effects of the combination antibiotics treatment on the anti-tumor activity^3^. We speculated that the alveolar macrophages be modulated when a selection of antibiotics was used. Unequal contribution by different species of commensal bacteria to the immunocompetence in the lungs was suggested, for example that neomycin sensitive bacteria associated with the induction of CD103^ +^ DCs in the lung and mLNs and their migration to the draining lymph node (LN)^27^. A low frequency of Proteobacteria and high frequencies of Firmicutes and Actinobacteria were observed in the upper respiratory tract of the Abt mice (Fig. 4d and e). When those recovered in the Abt mice by co-housing with WT mice, the alterations in alveolar macrophages were rescued (Fig. 4). Local microbiota-supplementary experiment in the Abt mice by nasal dripping upper respiratory tract microbiota from normal mice or some specific kind of bacterial such as Proteobacteria would further explain the microbiota regulation on the frequency, phenotype and function of alveolar macrophages.

Where the interaction between the microbiota in the upper respiratory tract and alveolar macrophages occur is an interesting problem. Alveolar macrophages from Abt mice tended to exhibit M2-associated behaviors due to the altered microbiota load and composition in the upper respiratory tract, as demonstrated by the expression of Arg1, CCL24, IL-13, IL-10, IL-6 and IL-1β (Fig. 4 and Supplemental Fig. 3). It was speculated that commensal-derived signals from the upper respiratory tract may provide necessary stimulation for alveolar macrophage polarization. Bacterial colonization of *S*. *aureus* could induce M2 alveolar macrophages in the environment and regulate the anti-viral immune responses^12^. The overgrowth of a commensal fungal *Candida* species in the gut induced M2 macrophage polarization in the lung through increased plasma concentrations of PGE2^6^. Noticeably, the gut microbiota is involved in systemic infections, such as influenza virus^27^, LCMV^37^, parasitic^38^ and bacterial infection^11^ and is involved in extra-intestinal autoimmune diseases including multiple sclerosis, type 1 diabetes, arthritis and allergic inflammation^6, 39^. Intestinal commensal-derived signals has been demonstrated to provide tonic immune stimulation that establishes the activation threshold of macrophages^37^. Thus, the distal effect of intestinal commensal microbiota should be taken account for the alveolar macrophages.

As for alveolar macrophages, they have a critical role in physiological immunosuppression for the lung immune homeostasis^40^. Alveolar macrophages reduced the number and maturation of lung DCs by regulating TGF-β in the lung environment, and also directly suppressed Th1 responses in the lungs, which contributed to the premetastatic niche^41^. In this study, alveolar macrophages from Abt mice showed much strongly suppressive functions on γδT17 immune response, resulting in a susceptibility to cancer progression compared with the normal mice (Fig. 6a,c and d). No differences in TGF-β expression were observed (Fig. 4a and Supplemental Fig. 3b), whereas much higher expression levels of the chemokine CCL24 were found in the alveolar macrophages from Abt mice (Fig. 4a,h, Supplemental Figs 3b and 5c). Furthermore, the tumor-promoting behavior of alveolar macrophages was dependent on CCL24 in the Abt mice (Fig. 6a). Until now, there has been limited research on the role of CCL24 in host tumor immune responses. In 2007, it was reported that high levels of CCL24 were strongly associated with the primary colorectal and liver metastatic tumors^20^. In contrast to the CCL24-mediated recruitment of CCR3^+^ immune cells, including eosinophils, basophils and Th2 cells during lung allergenic inflammation^10, 16^, high levels of CCL24 failed to induce the migration of CCR3^+^ immune cells in the tumor tissues. In our study, higher levels of CCL24 did not recruit lymphocytes into the lungs, but instead decreased the number of lymphocytes. CCL24 neutralization could promote immune cell infiltration in the lungs, particularly γδT17 cells (Fig. 6b,c and d, Supplement Fig. 6). These results revealed a novel feature of alveolar macrophage-derived CCL24 in the host tumor immune responses.

In summary, by using Abt mouse model we demonstrated the ability of commensal bacteria to maintain alveolar macrophages with a low level of CCL24 production, which was necessary for the normal anti-tumor response in the lung. Therefore, more attention should be paid to the deleterious effects of antibiotic treatment. The information from the gene microarray analysis in this study will help us to further explore the mechanisms by which microbiota regulates the expression of CCL24 in alveolar macrophages.

## Electronic supplementary material

Supplement Materials and Figures
