# Supplementary material for: Commensal microbiota maintains alveolar macrophages with a low level of CCL24 production to generate anti-metastatic tumor activity
Source: Sci Rep. 2017 Aug 7;7:7471. doi: 10.1038/s41598-017-08264-8 (PMC5547050; doi:10.1038/s41598-017-08264-8)

Commensal microbiota maintains alveolar macrophages with a low level of CCL24 production to generate anti-metastatic tumor activity

Min Cheng[1,2], Yongyan Chen[3], Liang Wang[1,2], Wen Chen[1,2], Ling Yang[4], Guodong Shen[1,2], Tingjuan Xu[1,2], Gan Shen[1,2], Zhigang Tian[3], Shilian Hu[1,2]

[1] Gerontology Institute of Anhui Province, Anhui Province Hospital, Anhui Medical University, Hefei, 230001, China;

[2] Anhui Provincial Key Laboratory of Tumor Immunotherapy and Nutrition Therapy, Hefei, 230001, China;

[3] Institute of Immunology and The CAS Key Laboratory of Innate Immunity and Chronic Disease, School of Life Science and Medical Center, University of Science and Technology of China, Hefei 230027, China

[4] School of Life Sciences, University of Science and Technology of China, Hefei 230027, China;

Address correspondence and reprint requests to Shilian Hu or Min Cheng. Gerontology Institute of Anhui Province, Anhui Province Hospital, Anhui Medical University, Hefei, 230001, China; E-mail: hushilian@126.com or chengmin@ustc.edu.cn.

Supplemental Materials and Methods

Supplemental Table 1. The monoclonal antibodies for FACS

Fluorescein	Antibody	Clone number	Manufacturer	Catalog number	Isotype control
FITC	anti-CD4	RM4-5	BD Pharmingen	553046	Rat IgG2α, κ
FITC	anti-CD69	H1.2F3	BD Pharmingen	553236	ArH IgG1, λ_3
FITC	anti-CD86	GL-1	BD Pharmingen	553691	Rat IgG2α, κ
FITC	anti-TCR-γδ	GL3	eBioscience	11-5711-81	ArH IgG
FITC	anti-F4/80	BM8	eBioscience	11-4801-81	Rat IgG2α, κ
PE	anti-CD3e	145-2C11	BD Pharmingen	553063	AH IgG1, κ
PE	anti-CD8α	53-6.7	BD Pharmingen	553033	Rat IgG2α, κ
PE	anti-CD16/32	2.4G2	BD Pharmingen	553145	Rat IgG2β, κ
PE	anti-CD11c	N418	eBioscience	12-0114-81	ArH IgG
PE	anti-CD127	A7R34	Biolegend	135010	Rat IgG2α, κ
PE	anti-MHC-II	M5/114.15.2	eBioscience	12-5321	Rat IgG2β, κ
PE	anti-TCR-γδ	GL3	eBioscience	12-5711-81	ArH IgG
PE	anti-IL-17A	TC11-18H10	BD Pharmingen	561020	Rat IgG1, κ
PerCP-CY5.5	anti-NK1.1	PK136	eBioscience	45-5941-80	MS IgG2α, κ
PerCP-CY5.5	anti-CCR7	4B12	eBioscience	45-1971	Rat IgG2α, κ
APC	anti-CD4	RM4-5	BD Pharmingen	561091	Rat IgG2α, κ
APC	anti-TCRβ	H57-597	Biolegend	109212	ArH IgG
APC	anti-TCR-γδ	GL3	eBioscience	17-5711-81	ArH IgG
APC	anti-IFN-γ	XMG1.2	BD Pharmingen	562018	Rat IgG1, κ
APC	anti-F4/80	BM8	eBioscience	17-4801-80	Rat IgG2α, κ

Quantitative Real-time PCR

mRNA was reverse transcribed into cDNA in a 200 µl reaction volume containing the following reagents: 15 µg of total mRNA, 5 µmol/L oligdT, 0.5 mmol/L dNTPs, 40 µl of 5×buffer, 10 mmol/L DTT, 400 units of RNase inhibitor, 2000 units of M-MLV and distilled water (ultrapure, DNase and RNase free). The RT reaction was performed at 37°C for 50 minutes, followed by heating at 70°C for 15 minutes. The standard 50 µl volume reaction contained 25 µl 2×PCR buffer, 2 µl cDNA template, 0.4 µmol/L forward and reverse primers. Quantitative real-time PCR was performed using Roche Light Cycler 480 II (Roche Diagnostics, Germany). PCR reactions were performed using a total of 45 cycles consisting of a 15 s melt at 95°C, followed by a 30 s annealing at 60°C, 30 s extension at 72°C. Each sample was analyzed in triplicate for each target gene.

Supplemental Table 2. The primers for each gene detected by real-time PCR

Gene	*Forward primer (5'-3')*	*Reverse primer (5'-3')*	*Amplicon length (bp)*	*Ref*
CD80	TTATCATCCTGGGCCTGGTC	GTGTCTGCAGATGGGTTTCC	169	*This work*
CD86	ACCAAATGCAGAGTGAAGGC	TCTTGGCCTATGAGTGTGCA	277	*This work*
TLR2	AGCATCCGAATTGCATCACC	ACCCCAGAAGCATCACATGA	175	*This work*
TLR4	AGATCTGAGCTTCAACCCCT	CCACAGCCACCAGATTCTCT	227	*This work*
CCL2	CCAGCAAGATGATCCCAATG	TACGGGTCAACTTCACATTC	249	*This work*
CCL3	GATTCCACGCCAATTCATCG	AGGCATTCAGTTCCAGGTCA	155	*This work*
CCL4	TTTCTCTTACACCTCCCGGC	AGCTGCTCAGTTCAACTCCA	181	*This work*
CCL5	CTGCTGCTTTGCCTACCTCT	TGTATTCTTGAACCCACTTCTTCT	158	*This work*
CCL8	GTCACCTGCTGCTTTCATGT	GAAGGTTCAAGGCTGCAGAA	213	*This work*
CCL9	TGCCCTCTCCTTCCTCATTC	GATAAAGATGATGCCCGGCC	247	*This work*
CCL11	GTCACTTCCTTCACCTCCCA	TTCTTGGGGTCAGCACAGAT	194	*This work*
CX3CL1	GTGCGACAAGATGACCTCAC	GCTTCTCAAACTTGCCACCA	212	*This work*
CXCL5	TTGCTTAACCGTAACTCCAA	ATTCCGCTTAGCTTTCTTTT	202	*This work*
CXCL9	TGCTACACTGAAGAACGGAGAT	TCAGGGTGCTTGTTGGTAAA	242	*This work*
CXCL10	TCATCCCTGCGAGCCTATCC	TGCGTGGCTTCACTCCAGTT	167	*This work*
CXCL11	TGGCAGAGATCGAGAAAGCT	ACGTTCCCAGGATGTCACAT	193	*This work*
CXCL16	GCCATTGTCTTCTTCCTCACT	CTCCCATGTCATCATCCATC	167	*This work*
IL-1b	GACCTTCCAGGATGAGGACA	AGGCCACAGGTATTTTGTCG	284	1
IL-12A	GATGACATGGTGAAGACGGC	AGGCACAGGGTCATCATCAA	227	*This work*
IL-15	GAGGCTGGCATTCATGTCTT	GCAATTCCAGGAGAAAGCAG	205	2
IL-18	GACAAAGAAAGCCGCCTCAA	GTGAAGTCGGCCAAAGTTGT	177	*This work*
IL-23A	CCAGCGGGACATATGAATCT	AGGCTCCCCTTTGAAGATGT	195	3
IFN-g	ACTGGCAAAAGGATGGTGAC	TGAGCTCATTGAATGCTTGG	237	4
TNF-a	CCACATCTCCCTCCAGAAAA	AGGGTCTGGGCCATAGAACT	259	5
IL-1R1	CAACGTGAGCTTCTTCGGAG	CGTGACGTTGCAGATCAGTT	244	*This work*
IDO2	GCCCTCAGACTTCCTCACTT	GTTTGTCAGCACCAGGTCAG	273	*This work*
STAT1	CCTGGAGGTCTTTGTTCCCT	TCAACACCTCTGAGAGCTGG	185	*This work*
NOS2	CCCCGCTACTACTCCATCAG	CCACTGACACTTCGCACAAA	185	*This work*
CD163	CACTCTTGGTTTGTGGAGCC	TCCTTCTCAGTGTGCCTCTG	208	*This work*
TLR1	AGGATGTGACTGACCCCTTG	CATTCCTGAGGTCCCTGCTA	157	*This work*
TLR8	GGACCACTGCACTGCTACTA	AGGACAGCAGACAAAAGCAC	259	*This work*
CCL1	GATGGGCTCCTCCTGTCCTG	CTGGGATGGGAAGGTGGCTC	157	*This work*
CCL17	TCACTTCAGATGCTGCTCCT	CAGTCAGAAACACGATGGCA	193	*This work*
CCL22	ACCCTCTGCCATCACGTTTA	GGCAGAAGAATAGGGCTTGC	220	*This work*
CCL24	CTCCTTCTCCTGGTAGCCTG	ATGGCCCTTCTTGGTGATGA	183	*This work*
CXCL13	GTTGTCGGTCTAAACATCATAG	AATTCAGAGCAGGGATAAAA	268	*This work*
IL-4	AACGAGGTCACAGGAGAAGG	TCTGCAGCTCCATGAGAACA	187	*This work*
IL-6	AACGATGATGCACTTGCAGA	GGAAATTGGGGTAGGAAGGA	276	6
IL-10	GGTGAGAAGCTGAAGACCCT	TGTCTAGGTCCTGGAGTCCA	248	7
IL-13	GCAGCATGGTATGGAGTGTG	TGGCGAAACAGTTGCTTTGT	226	*This work*
TGF-b3	GTCACACCTTTCAGCCCAAT	GATCCTGCCGGAAGTCAATA	297	8
IL-1R2	CTAGTGTGCCCTGACCTGAA	GATGGGTTCCGTGGTTGTTC	261	*This work*

IL-1Rn	CAAGCTGTGCCTGTCTTGTG	GACGGTCAGCCTCTAGTGTT	200	*This work*
Arg1	GCTGGGAAGGAAGAAAAGGC	TGCCGTGTTCACAGTACTCT	232	*This work*
IGF1	ACTGGAGATGTACTGTGCCC	CAAAGGATCCTGCGGTGATG	253	*This work*
RETNLA	TCCCTTCTCATCTGCATCTCC	CCCAAGATCCACAGGCAAAG	238	*This work*
MRC1	AAGGAAACCATGGACAACGC	ACTTTGCTCCCATCCATCCA	188	*This work*
MSR1	GAAAAGGGAGACAGAGGGCT	TCTTGATCCGCCTACACTCC	207	*This work*
Chi3l3	TGGAATTGGTGCCCCTACAA	CCACGGCACCTCCTAAATTG	239	*This work*
Chi3l4	GGATGGAAGTTTGGACCTGC	AGTGAGTAGCAGCCTTGGAA	252	*This work*
TGM2	CAAGCAAAACCGCAAACTGG	GGAAGTTCACCACCAGCTTG	215	*This work*
CLEC7A	CTGGGAGGATGGATCAGCAT	TTGTTGGTAGTGGTGGTGGT	249	*This work*
Amac1	CTCGAGAAGGTTCCAGGGAA	GCGATGGTGATGTTTGGAGG	213	*This work*
RBP4	TTCTGTGGACGAGAAGGGTC	GTGCCATCCAGATTCTGCAG	249	*This work*
NPY	TCGTGTGTTTGGGCATTCTG	GGCGTTTTCTGTGCTTTCCT	203	*This work*
Bex1	CCTGGTGGTGAGCATCTCTA	CTGGCTCCCTTCTGATGGTA	160	*This work*
MMP12	TTTGGAGCTCACGGAGACTT	GCGAAATGTGCTGGGGTTAA	255	*This work*
H2-M2	ATGAGGCTGAGAATCCGAGG	TCCAATGTAGCAGCCAGTCA	200	*This work*
Car6	TGACGGCACTGAGTTCATCT	GGCAGAAATGATGTCGCTGT	250	*This work*
Timp1	TATGCCCACAAGTCCCAGAA	ACTCTCCAGTTTGCAAGGGA	210	*This work*
Trim6	TGCAAGGAGGATGGGAAGTT	CTCCACCTGACTCTTCCAGG	210	*This work*
β-actin	TGACGTTGACATCCGTAAAGACC	CTCAGGAGGAGCAATGATCTTGA	148	9

Determination of adoptively transferred alveolar macrophages in the lungs

Purified alveolar macrophages (F4/80^{hi} CD11c^{hi}) (2×10^{5} cells in 50 μl PBS) were adoptively transferred intranasally (i.n.) into the recipient mouse. The same volume of PBS alone was used as the control. After 12h of the cell transfer, the recipient mice were sacrificed and the lung MNCs were isolated, and then directly detected by flow cytometry to show the frequency of F4/80^{hi} CD11c^{hi} cells. According to the total number of MNCs, the number of transferred macrophages in the lungs of the recipient mouse was calculated.

Supplemental Table 3. The up-regulated and down-regulated genes with biological functions in alveolar macrophages of Abt mice

Gene Name	Gene Symbol	Fold change	FDR
**Membrane and transmembrane protein**			
sel-1 suppressor of lin-12-like 3 (C. elegans)	SEL1L3	10.89	0.134
transmembrane protein 26	TMEM26	9.56	0.197
acyl-CoA wax alcohol acyltransferase 1	AWAT1	6.41	0.154
plakophilin 2	PKP2	4.95	0.097
5-hydroxytryptamine (serotonin) receptor 2B	HTR2B	4.84	0.178
synaptogyrin 1	SYNGR1	4.80	0.104
solute carrier family 6 (neurotransmitter transporter, creatine), member 8	SLC6A8	4.08	0.141
solute carrier family 36 (proton/amino acid symporter), member 2	SLC36A2	4.04	0.154
purinergic receptor P2Y, G-protein coupled 1	P2RY1	3.99	0.082
ectonucleoside triphosphate diphosphohydrolase 3	ENTPD3	3.87	0.168
transmembrane protein 171	TMEM171	3.01	0.213
transmembrane protein 37	TMEM37	2.95	0.158
arginine vasopressin receptor 2	AVPR2	2.91	0.177
potassium voltage-gated channel, Isk-related subfamily, gene 4	KCNE4	2.89	0.250
RAB38, member of RAS oncogene family	RAB38	2.82	0.166
zinc finger, DHHC domain containing 14	ZDHHC14	2.81	0.314
mcf.2 transforming sequence-like	MCF2L	2.76	0.196
solute carrier family 6 (neurotransmitter transporter, betaine/GABA), member 12	SLC6A12	2.74	0.081
ArfGAP with SH3 domain, ankyrin repeat and PH domain 2	ASAP2	2.73	0.095
solute carrier family 30 (zinc transporter), member 4	SLC30A4	2.61	0.088
interleukin 7 receptor	IL7R	2.53	0.209
flavin containing monooxygenase 4	FMO4	2.44	0.321
gap junction protein, alpha 10	GJA10	2.39	0.368
cytochrome P450, family 4, subfamily a, polypeptide 31	CYP4A31	2.37	0.195
brain-specific angiogenesis inhibitor 1-associated protein 2	BAIAP2	2.36	0.174
calcium homeostasis modulator 2	CALHM2	2.31	0.320
X Kell blood group precursor related X linked	XKRX	2.31	0.296
solute carrier family 22 (organic cation transporter), member 4	SLC22A4	2.29	0.161
membrane bound O-acyltransferase domain containing 1	MBOAT1	2.23	0.319
guanine nucleotide binding protein, alpha O	GNAO1	2.22	0.332
epidermal growth factor receptor	EGFR	2.22	0.385
prostaglandin I receptor (IP)	PTGIR	2.13	0.164
ATP-binding cassette, sub-family A (ABC1), member 8b	ABCA8B	2.12	0.392
regulating synaptic membrane exocytosis 4	RIMS4	2.06	0.435
chemokine (C-X-C motif) receptor 1	CXCR1	2.03	0.101
ribosomal protein S6 kinase polypeptide 1	RPS6KC1	2.02	0.124
toll-like receptor 12	TLR12	2.01	0.324
testis specific 10	TSGA10	2.01	0.157
MRV integration site 1	MRVI1	-2.04	0.197
membrane associated guanylate kinase, WW and PDZ domain containing 1	MAGI1	-2.08	0.270
oxysterol binding protein 2	OSBP2	-2.08	0.369
aquaporin 3	AQP3	-2.10	0.328
aquaporin 5	AQP5	-2.13	0.415
transmembrane protein 204	TMEM204	-2.14	0.297
armadillo repeat gene deleted in velo-cardio-facial syndrome	ARVCF	-2.16	0.093
claudin 5	CLDN5	-2.24	0.436

vomeronasal 2, receptor33	VMN2R33	-2.61	0.083
neuropeptide Y receptor Y1	NPY1R	-2.63	0.300
chloride intracellular channel 5	CLIC5	-2.76	0.298
SH3-domain binding protein 4	SH3BP4	-3.06	0.224
vomeronasal 2, receptor, pseudogene 57	VMN2R-PS 57	-6.41	0.120
**Cytokines and chemokines**			
chemokine (C-C motif) ligand 24	CCL24	36.24	0.096
gremlin 1	GREM1	7.81	0.167
chemokine (C-C motif) ligand 8	CCL8	4.50	0.293
chemokine (C-X-C motif) ligand 9	CXCL9	4.10	0.493
chemokine (C-C motif) ligand 17	CCL17	3.06	0.209
colony stimulating factor 1 (macrophage)	CSF1	2.86	0.226
interleukin 7	IL7	2.04	0.084
T cell lymphoma invasion and metastasis 2	TIAM2	2.02	0.164
**Immune response and inflammatory response**			
arginase	ARG1	59.45	0.089
histocompatibility 2, M region locus 2	H2-M2	10.97	0.089
serum amyloid A 3	SAA3	6.22	0.332
serine (or cysteine) peptidase inhibitor, clade A, member 3G	SERPINA3 G	5.01	0.253
myomesin family, member 3	MYOM3	4.43	0.175
T cell immunoreceptor with Ig and ITIM domains	TIGIT	4.00	0.413
Cd200 receptor 2	CD200R2	3.27	0.161
complement component 4B (Chido blood group)	C4B	3.23	0.194
cDNA sequence BC055004	BC055004	3.01	0.246
SLAM family member 7	SLAMF7	2.34	0.177
neutrophilic granule protein	NGP	2.34	0.336
3'-phosphoadenosine 5'-phosphosulfate synthase 2	PAPSS2	2.21	0.159
B cell linker	BLNK	2.19	0.207
collectin sub-family member 12	COLEC12	2.17	0.160
SLAM family member 8	SLAMF8	2.11	0.307
triggering receptor expressed on myeloid cells-like 2	TREML2	2.09	0.176
Fc receptor-like 1	FCRL1	2.03	0.242
T cell immunoglobulin and mucin domain containing 4	TIMD4	2.03	0.157
phospholipase A2, group VII	PLA2G7	2.03	0.201
pregnancy-specific glycoprotein 25	PSG25	2.00	0.204
RIKEN cDNA A630038E17 gene	A630038E1 7RIK	-2.01	0.146
coagulation factor II (thrombin) receptor-like 3	F2RL3	-2.34	0.089
immunoglobulin heavy chain (J558 family)	IGH-VJ558	-2.49	0.165
**Growth, proliferation and differentiation**			
retinol binding protein 4, plasma	RBP4	133.14	0.080
brain expressed gene 1	BEX1	30.05	0.100
inhibin beta-A	INHBA	4.75	0.115
neuregulin 1	NRG1	4.44	0.212
insulin-like growth factor 1	IGF1	2.64	0.087
palmdelphin	PALMD	2.58	0.238
retinoic acid receptor, beta	RARB	2.39	0.088
NK6 homeobox 2	NKX6-2	2.39	0.402
diencephalon/mesencephalon homeobox 1	DMBX1	2.37	0.319
PTK2 protein tyrosine kinase 2	PTK2	2.33	0.138
sphingosine kinase 1	SPHK1	2.28	0.292

prion protein	PRNP	2.27	0.225
integrin beta 3	ITGB3	2.20	0.173
B cell scaffold protein with ankyrin repeats 1	BANK1	2.11	0.261
predicted gene 98	GM98	2.09	0.136
myeloid cell nuclear differentiation antigen	MNDA	2.03	0.433
peripheral myelin protein 22	PMP22	2.02	0.268
inositol polyphosphate 5-phosphatase J	INPP5J	2.00	0.224
ephrin A1	EFNA1	-2.21	0.243
caveolin 3	CAV3	-2.29	0.239
**Signal**			
laminin gamma 3	LAMC3	2.48	0.240
glypican 1	GPC1	2.44	0.244
mucin 19	MUC19	2.39	0.210
phospholipase A2, group IID	PLA2G2D	2.31	0.164
ADAM-like, decysin 1	ADAMDEC 1	2.24	0.308
anterior gradient 2 (Xenopus laevis)	AGR2	2.24	0.368
family with sequence similarity 198, member B	FAM198B	2.21	0.162
laminin, gamma 2	LAMC2	2.19	0.157
ectonucleotide pyrophosphatase/phosphodiesterase 5	ENPP5	2.18	0.200
odd Oz/ten-m homolog 4 (Drosophila)	ODZ4	2.17	0.266
UDP-Gal:betaGlcNAc beta 1,4-galactosyltransferase, polypeptide 6	B4GALT6	2.14	0.300
eosinophil-associated, ribonuclease A family, member 5	EAR5	2.13	0.156
G protein-coupled receptor, family C, group 5, member B	GPRC5B	2.12	0.244
leucine-rich alpha-2-glycoprotein 1	LRG1	2.05	0.161
eosinophil-associated, ribonuclease A family, member 7	EAR7	2.02	0.089
latrophilin 1	LPHN1	2.01	0.317
mesothelin-like	MSLNL	-2.28	0.265
C1q and tumor necrosis factor related protein 4	C1QTNF4	-2.60	0.314
CD163 molecule-like 1	CD163L1	-4.30	0.111
glycosyltransferase 6 domain containing 1	GLT6D1	-4.71	0.177
**Enzyme inhibitor activity and regulation of kinase activity**			
tissue inhibitor of metalloproteinase 1	TIMP1	6.47	0.180
serine (or cysteine) peptidase inhibitor, clade B, member 2	SERPINB2	3.69	0.286
serine (or cysteine) peptidase inhibitor, clade A, member 3F	SERPINA3F	2.90	0.320
predicted gene 11428	GM11428	2.36	0.165
serine peptidase inhibitor, Kazal type 2	SPINK2	2.28	0.202
serine (or cysteine) peptidase inhibitor, clade B (ovalbumin), member 10	SERPINB10	2.17	0.137
stefin A1	STFA1	2.10	0.266
**Angiogenesis and vasculature development**			
alanyl (membrane) aminopeptidase	ANPEP	4.49	0.088
heparin-binding EGF-like growth factor	HBEGF	3.89	0.202
platelet derived growth factor, alpha	PDGFA	3.51	0.197
endothelin 1	EDN1	2.99	0.358
phosphatidic acid phosphatase type 2B	PPAP2B	2.72	0.166
angiopoietin 2	ANGPT2	2.36	0.213
podoplanin	PDPN	2.06	0.151
**Metalloprotease**			
matrix metallopeptidase 12	MMP12	13.64	0.090
matrix metallopeptidase 13	MMP13	2.68	0.247
matrix metallopeptidase 3	MMP3	2.11	0.330

Reference

1 Brambilla, R. *et al.* Transgenic inhibition of astroglial NF-kappaB protects from optic nerve damage and retinal ganglion cell loss in experimental optic neuritis. *J Neuroinflammation* **9**, 213, doi:10.1186/1742-2094-9-213 (2012).

2 Novy, P., Huang, X., Leonard, W. J. & Yang, Y. Intrinsic IL-21 signaling is critical for CD8 T cell survival and memory formation in response to vaccinia viral infection. *J Immunol* **186**, 2729-2738, doi:10.4049/jimmunol.1003009 (2011).

3 Wang, J., Ma, J., Charboneau, R., Barke, R. & Roy, S. Morphine inhibits murine dendritic cell IL-23 production by modulating Toll-like receptor 2 and Nod2 signaling. *The Journal of biological chemistry* **286**, 10225-10232, doi:10.1074/jbc.M110.188680 (2011).

4 Salcedo, R. *et al.* IL-27 mediates complete regression of orthotopic primary and metastatic murine neuroblastoma tumors: role for CD8+ T cells. *J Immunol* **173**, 7170-7182, doi:173/12/7170 (2004).

5 Wang, M. E. *et al.* Curcumin protects against thioacetamide-induced hepatic fibrosis by attenuating the inflammatory response and inducing apoptosis of damaged hepatocytes. *The Journal of nutritional biochemistry* **23**, 1352-1366, doi:10.1016/j.jnutbio.2011.08.004 (2012).

6 Chae, M. J. *et al.* Chemical inhibitors destabilize HuR binding to the AU-rich element of TNF-alpha mRNA. *Experimental & molecular medicine* **41**, 824-831, doi:10.3858/emm.2009.41.11.088 (2009).

7 Ge, R. T. *et al.* Insulin-like growth factor-1 endues monocytes with immune suppressive ability to inhibit inflammation in the intestine. *Sci Rep* **5**, 7735, doi:10.1038/srep07735 (2015).

8 Huen, S. C., Moeckel, G. W. & Cantley, L. G. Macrophage-specific deletion of transforming growth factor-beta1 does not prevent renal fibrosis after severe ischemia-reperfusion or obstructive injury. *Am J Physiol Renal Physiol* **305**, F477-484, doi:10.1152/ajprenal.00624.2012 (2013).

9 Mathew, R. *et al.* Meprin-alpha in chronic diabetic nephropathy: interaction with the renin-angiotensin axis. *Am J Physiol Renal Physiol* **289**, F911-921, doi:00037/ajprenal.00037 (2005).

Supplemental Figure Legends

Supplemental Figure 1. Alveolar macrophages exhibited a distinct transcriptome profile in the Abt mice. The mice in Abt group were given antibiotics for five weeks. Purified alveolar macrophages (F4/80^{hi} CD11c^{hi}) were analyzed by GeneChip. (a) Principal component analysis (PCA) was performed to assess the macrophage transcriptomes of the Abt group (red) and the control group (black). There were two samples per group. (b) Hierarchical clustering based on the Euclidean distance of the Abt and control samples. (c) The pie chart showed the distribution of DEGs in Abt group compared with the control. Indistinguishable from the background signal, unknown and duplicated genes were filtered for the analysis.

Supplemental Figure 2. Phenotypes of alveolar macrophages in Abt mice. C57BL/6 mice in the Abt group were given 1 g/L ampicillin, 0.5 g/L vancomycin, 1 g/L neomycin sulfate, and 1 g/L metronidazole in their drinking water for five weeks. The MNCs were isolated from lungs in each group and analyzed by FACS. Alveolar macrophages (F4/80^{hi} CD11c^{hi}) were gated for the analysis of the indicated phenotypes. There were five mice per group.

Supplemental Figure3. M1 and M2 specific gene expression in alveolar macrophages from Abt mice compared with normal mice. The mice in Abt group were given antibiotics for five weeks. Purified alveolar macrophages (F4/80^{hi} CD11c^{hi}) were analyzed. The mRNA expression levels of M1 specific genes (a) and M2 specific genes (b) in sorted macrophages (F4/80^{hi} CD11c^{hi}) were measured by real-time PCR (n=3). The data are shown as the mean ± SEM. *$p < 0.05$, compared with the control group.

Supplemental Figure 4. Differentially modulated genes in alveolar macrophages in Abt mice challenged with B16/F10 melanoma. The mice were given antibiotics for five weeks and then challenged with B16/F10 cells (1×10^5 cells/mouse, i.v.). On day 17 after the B16/F10 challenge,

purified alveolar macrophages ($F4/80^{hi}$ $CD11c^{hi}$) were analyzed by GeneChip (2 samples/group, 15 mice/sample). (a) Protein-protein interaction (PPI) networks of the DEGs identified in Abt group challenged with B16/F10 melanoma compared with the control. The red nodes indicate the up-regulated DEGs and the blue nodes indicate the down-regulated DEGs. Proteins associated with each other are linked by an edge. The degree of color represents the fold change of DEGs in reference to the scaleplate. (b) The list of DEGs was converted to Entrez-IDs for GO analysis with R 3.2.3 using the library GOstats 2.34.0 and the R Bioconductor genomewide mouse annotations from the package org.Mm.eg.db (version 3.3.0). The results were sorted by p-value ($p < 0.01$).

Supplemental Figure 5. The procedures for the adoptive transfer of alveolar macrophages and CCL24 neutralization in Abt mice challenged with B16/F10 melanoma. The mice were given antibiotics for five weeks and then challenged with B16/F10 cells (1×10^5 cells/mouse, i.v.). The mice were treated with antibiotics for the entire experimental period. The lungs were analyzed on day 17 after the B16/F10 melanoma challenge. (a) Purified alveolar macrophages from donor water-mice were adoptively transferred into the recipient Abt mice one day prior to the B16/F10 challenge, followed by additional transfers every week. Anti-CCL24 antibody was injected into the Abt mice one day prior to B16/F10 challenge, and additional injections were performed every week. (b) Purified alveolar macrophages from donor water-mice for the transfer.

Supplemental Figure 6. The adoptively transferred alveolar macrophages could reach the lungs of the recipient mice. The lung MNCs were isolated from the normal 9- to 10-week-old female C57BL/6 mice, and labeled with the FITC-anti-F4/80 and PE-anti-CD11c. Then the alveolar macrophages ($F4/80^{hi}$ $CD11c^{hi}$) were sorted by flow cytometry (BD Aria II). Purified alveolar macrophages (2×10^5 cells in 50 μl PBS) were adoptively transferred intranasally (i.n.) into the recipient mouse. The same volume of PBS alone was used as the control. After 12h of the cell transfer, the recipient mice were sacrificed and the lung MNCs were isolated, and then directly

detected by flow cytometry. $F4/80^{hi}$ $CD11c^{hi}$ cells were the transferred alveolar macrophages. The frequency and absolute number were shown. There were three mice in each group.

Supplemental Figure 7. Frequency and number of NK, NKT, conventional T cells in the lung of Abt mice after the adoptive transfer of normal alveolar macr

ophages or CCL24 neutralization. The mice were given antibiotics for five weeks and then challenged with B16/F10 cells (1×10^5 cells/mouse, i.v.). Alveolar macrophages transfer and CCL24 neutralization were performed in the Abt mice. The mice were treated with antibiotics for the entire experimental period. The isolated MNCs were analyzed by FACS. The lymphocytes were gated by FSC and SSC. The frequency and the absolute number of each lymphocyte subset in the lung are shown. There were six mice in each group.The data are shown as the mean ± SEM. * $p < 0.05$ compared with the control group.

Supplemental Figure 8. Anti-tumor immune responses in spleen of Abt mice challenged with B16/F10 melanoma. The mice were given antibiotics for five weeks and then challenged with B16/F10 cells (1×10^5 cells/mouse, i.v.). Alveolar macrophage transfer and CCL24 neutralization were performed in Abt mice as described in Supplemental Figure 5A. The mice were treated with antibiotics for the entire experimental period. (a) The total number of MNCs in the spleen on day 17 after the B16/F10 melanoma challenge. (b) The frequency and the absolute number of each lymphocyte subset in the spleen are shown. The isolated MNCs were analyzed by FACS and the lymphocytes were gated by FSC and SSC. (c) The frequency and the absolute number of IL-$17A^+$ γδT cells or IFN-γ^+ γδT cells among γδT cells in the spleen are shown. There were six mice in each group. Data are shown as mean ± SEM. * $p < 0.05$ compared with the control group.

***Cheng M*, supplemental Figure 1**

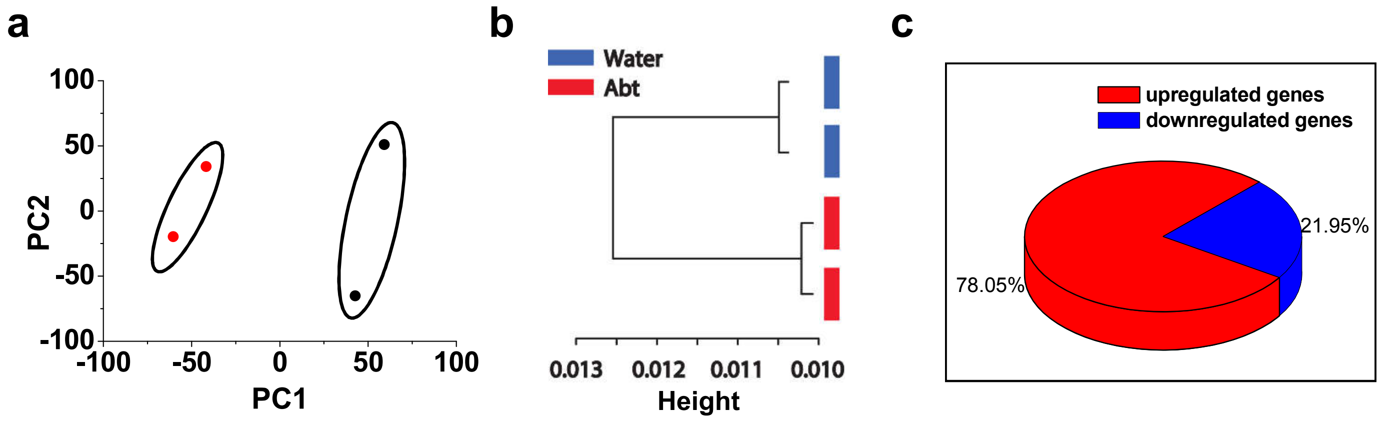

***Cheng M*, supplemental Figure 2**

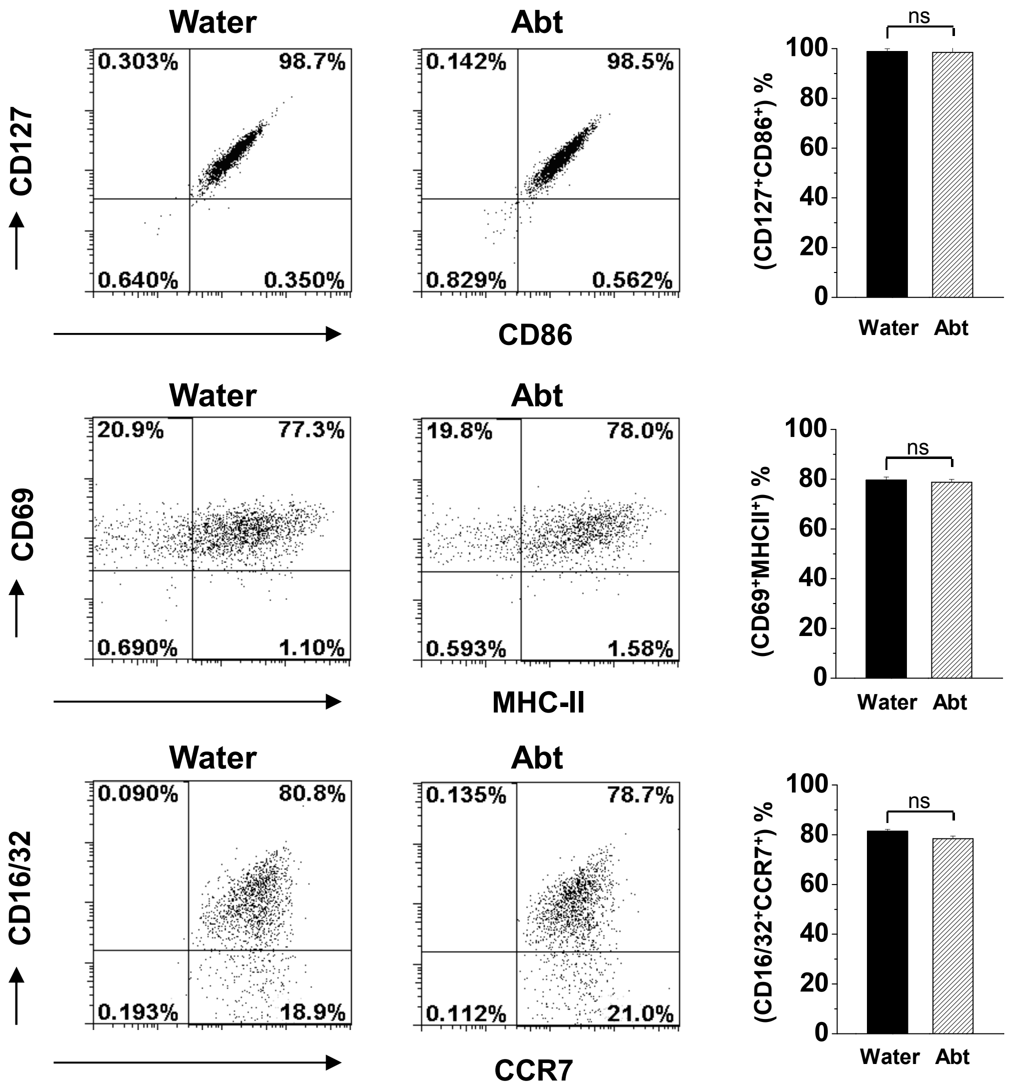

***Cheng M*, supplemental Figure 3**

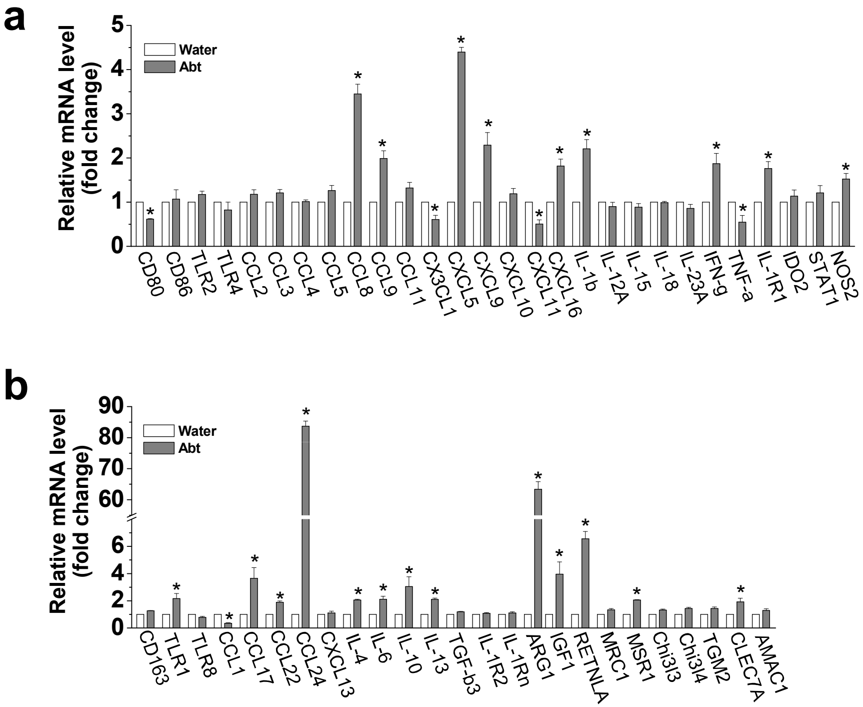

Cheng M, **supplemental Figure 4**

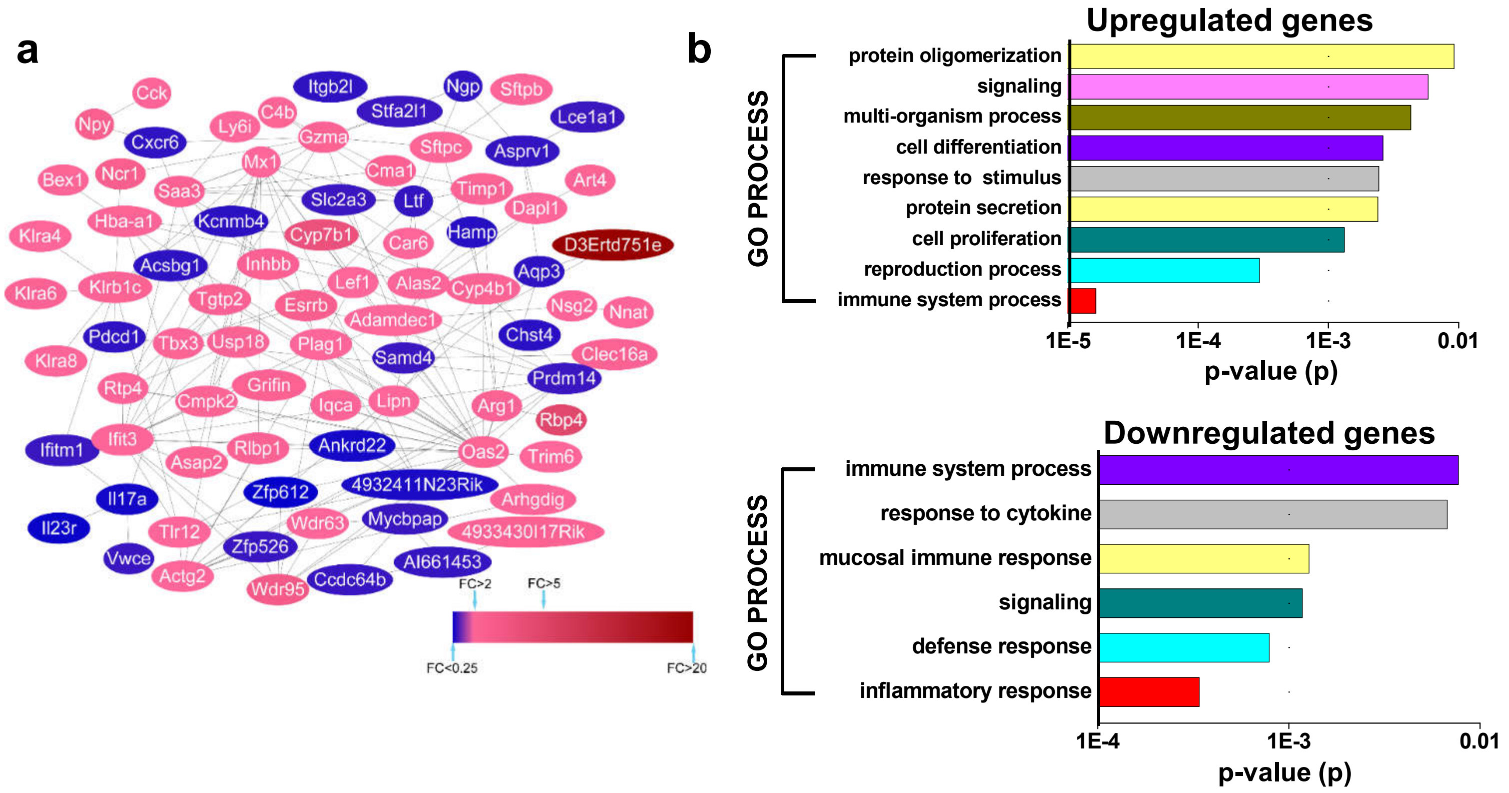

Cheng M, supplemental Figure 5

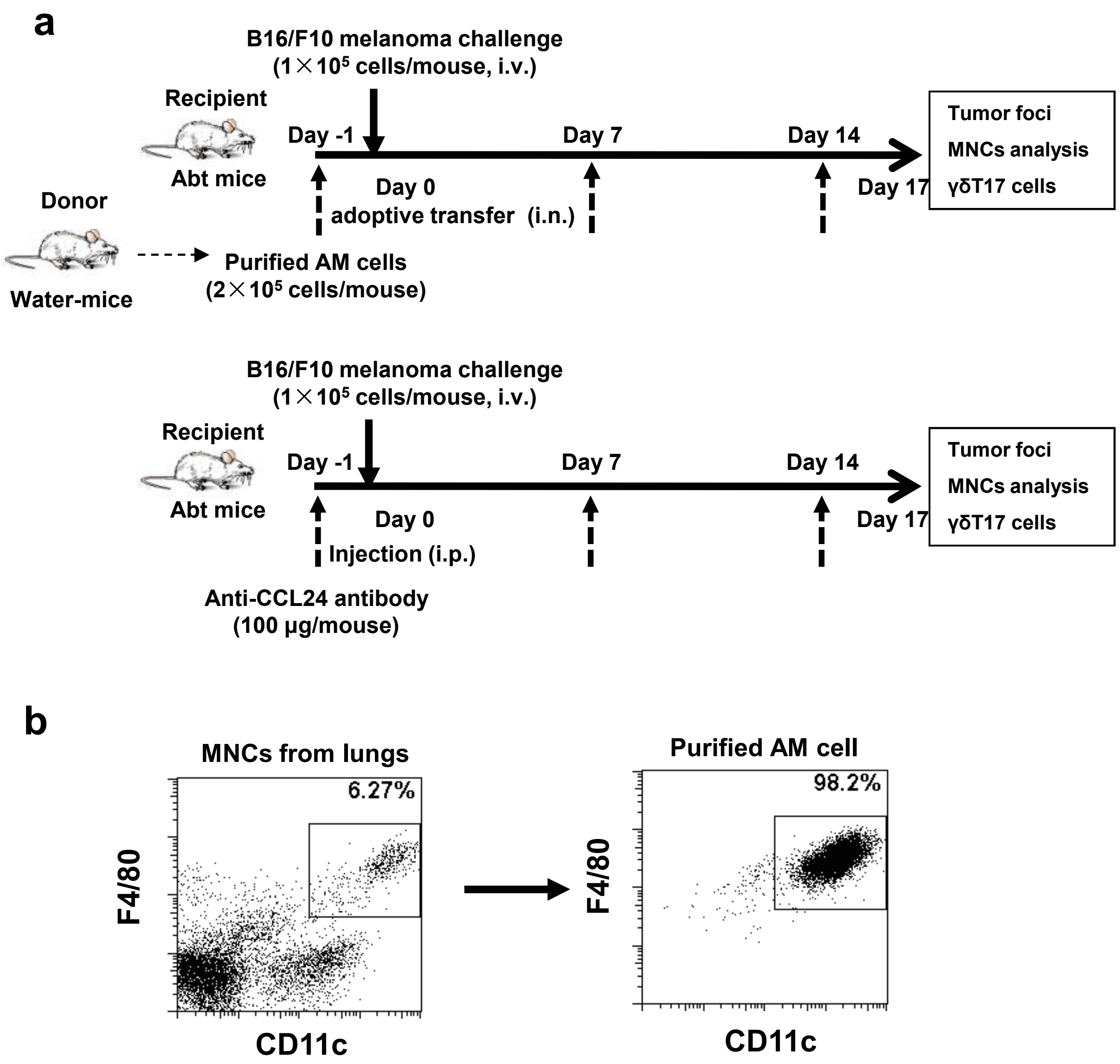

Cheng M, **supplemental Figure 6**

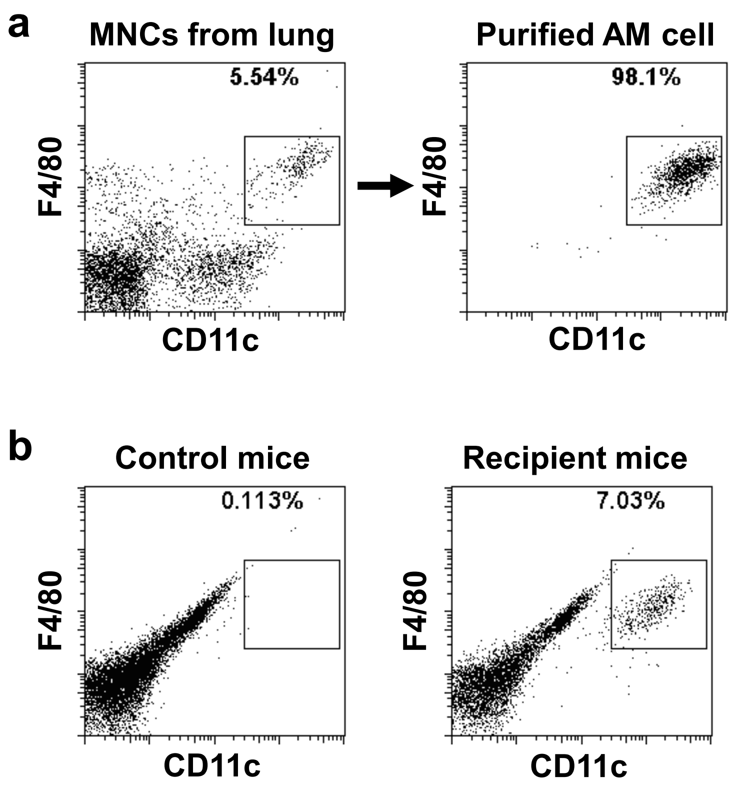

Cheng M, **supplemental Figure 7**

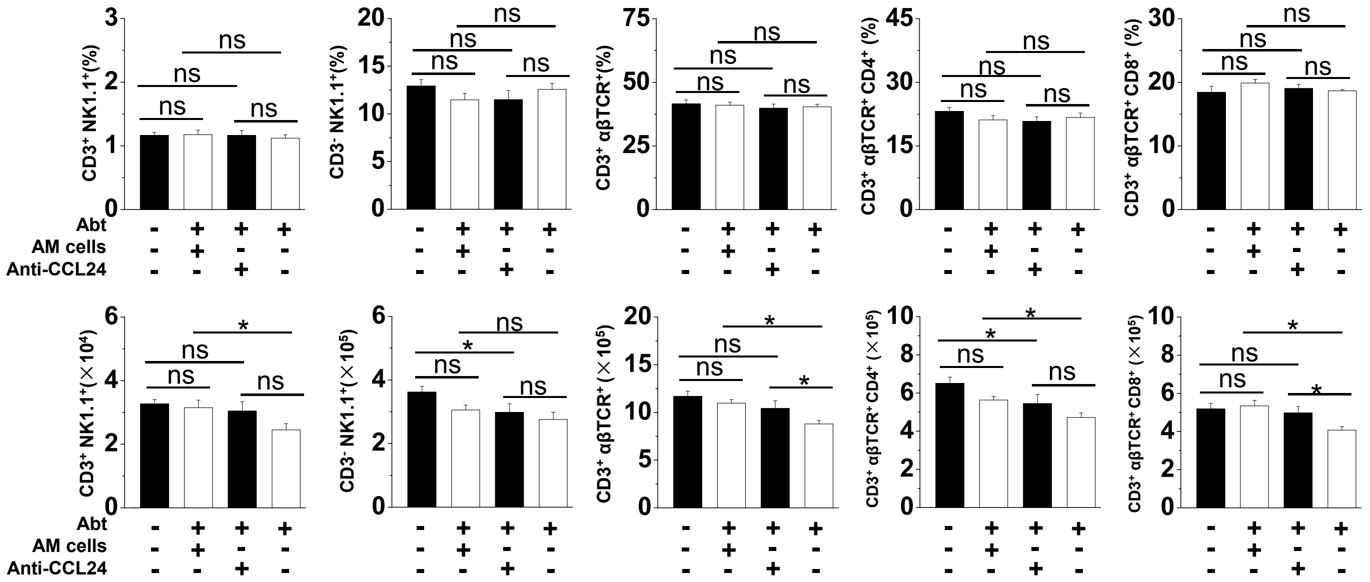

Cheng M, **supplemental Figure 8**

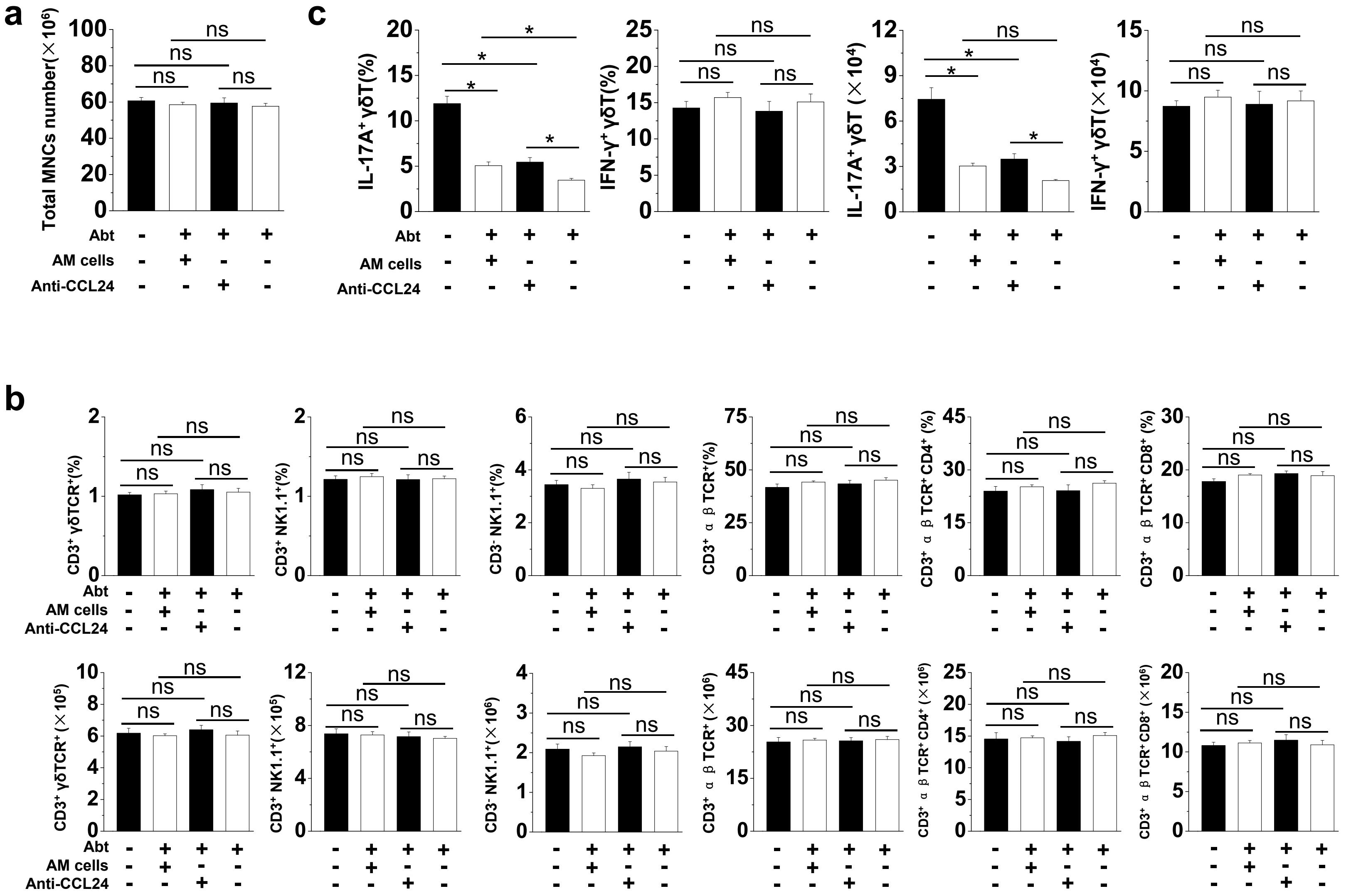

Supplement: Supplementary file 1 — Supplement Materials and Figures [file 41598_2017_8264_MOESM1_ESM.pdf]
